# CD4/CD8 ratio and CD8+ T-cell count as prognostic markers for non-AIDS mortality in people living with HIV. A systematic review and meta-analysis

**DOI:** 10.3389/fimmu.2024.1343124

**Published:** 2024-02-01

**Authors:** Raquel Ron, Javier Martínez-Sanz, Sabina Herrera, Luis Ramos-Ruperto, Alejandro Díez-Vidal, Talía Sainz, Noelia Álvarez-Díaz, Andrea Correa-Pérez, Alfonso Muriel, Jesús López-Alcalde, José A. Pérez-Molina, Santiago Moreno, Sergio Serrano-Villar

**Affiliations:** ^1^ Department of Infectious Diseases, Hospital Universitario Ramón y Cajal and Instituto Ramón y Cajal de Investigación Sanitaria (IRYCIS), Madrid, Spain; ^2^ Universidad de Alcalá (UAH), Madrid, Spain; ^3^ CIBER de Enfermedades Infecciosas (CIBERINFEC), Instituto de Salud Carlos III, Madrid, Spain; ^4^ Infectious Diseases Department, Hospital Clinic, Barcelona, Spain; ^5^ HIV Unit, Internal Medicine Department, Hospital Universitario La Paz, Madrid, Spain; ^6^ Pediatric Tropical and Infectious Diseases, Hospital la Paz and La Paz Research Institute (IdiPAZ), Center for Biomedical Research in Infectious Diseases (CIBERINFEC), Universidad Autoónoma de Madrid, UAM, Madrid, Spain; ^7^ Medical Library, Hospital Universitario Ramón y Cajal, Instituto Ramón y Cajal de Investigación Sanitaria (IRYCIS), Madrid, Spain; ^8^ Faculty of Medicine, Universidad Francisco de Vitoria (UFV), Madrid, Spain; ^9^ Pharmacy and Medical Devices Department. Hospital Central de la Defensa Gómez Ulla, Madrid, Spain; ^10^ Clinical Biostatistics Unit, Hospital Universitario Ramón y Cajal, Instituto Ramón y Cajal de Investigación Sanitaria (IRYCIS), Centro de Investigación Biomédica en Red. Epidemiología y Salud Pública (CIBERESP), Madrid, Spain; ^11^ Institute for Complementary and Integrative Medicine, University Hospital Zurich and University of Zurich, Zurich, Switzerland

**Keywords:** HIV, CD4/CD8 ratio, mortality, comorbidities, non-AIDS events

## Abstract

**Background:**

In people living with HIV (PLHIV), the CD4/CD8 ratio has been proposed as a useful marker for non-AIDS events. However, its predictive ability on mortality over CD4 counts, and the role of CD8+ T-cell counts remain controversial.

**Methods:**

We conducted a systematic review and meta-analysis of published studies from 1996 to 2023, including PLHIV on antiretroviral treatment, and reporting CD4/CD8 ratio or CD8+ counts. The primary outcome was non-AIDS mortality or all-cause mortality. We performed a standard random-effects pairwise meta-analysis comparing low versus high CD4/CD8 ratio with a predefined cut-off point of 0.5. (CRD42020170931).

**Findings:**

We identified 2,479 studies for screening. 20 studies were included in the systematic review. Seven studies found an association between low CD4/CD8 ratio categories and increased mortality risk, with variable cut-off points between 0.4-1. Four studies were selected for meta-analysis, including 12,893 participants and 618 reported deaths. Patients with values of CD4/CD8 ratio below 0.5 showed a higher mortality risk (OR 3.65; 95% CI 3.04 - 4.35; I2 = 0.00%) compared to those with higher values. While the meta-analysis of CD8+ T-cell counts was not feasible due to methodological differences between studies, the systematic review suggests a negative prognostic impact of higher values (>1,138 to 1,500 cells/uL) in the long term.

**Conclusions:**

Our results support the use of the CD4/CD8 ratio as a prognostic marker in clinical practice, especially in patients with values below 0.5, but consensus criteria on ratio timing measurement, cut-off values, and time to event are needed in future studies to get more robust conclusions.

**Systematic review registration:**

https://www.crd.york.ac.uk/prospero/display_record.php?ID=CRD42020170931, identifier CRD42020170931.

## Introduction

The optimization of antiretroviral treatment (ART) has facilitated a marked increase in life expectancy for individuals living with HIV (PLHIV) (1, 2). However, this enhancement in survival has also led to an upsurge in the prevalence of comorbidities and non-AIDS-related diseases (3–5). Despite the high rates of virological suppression obtained with current drugs, a percentage of patients do not achieve complete immune restoration. This is represented by the persistence of a decreased CD4/CD8 ratio, partially attributable to a high CD8+ T-cell count (6–8). PLHIV with a low CD4/CD8 ratio demonstrates heightened inflammation and immunosenescence, even under successful ART and adequate CD4^+^ count recovery (>500 cells/μL) (7, 9), and chronic HIV infection is characterized by an increase in CD8+ T-cell count along with functionality changes (10).

Therefore, the CD4/CD8 ratio has evolved as a valuable proxy for immune dysfunction among PLHIV. Its ease of monitoring in standard clinical settings, along with its correlation to markers of immunosenescence and inflammation enhances its utility at an individual level (5, 6, 11). In PLHIV on ART, the ratio correlates with a spectrum of comorbidities, such as cerebrovascular disease (12), chronic obstructive pulmonary disease (13), chronic kidney disease (14), and non-AIDS related cancers (15–17), thereby highlighting its role as a predictive marker for severe non-AIDS events (SNAEs) such as non-AIDS related mortality. However, the relevance of the CD4/CD8 ratio over isolated CD4+ or CD8+ count and its potential use as a predictor of mortality remains unclear. While some studies have reported a higher risk of non-AIDS events and mortality in patients with a CD4/CD8 ratio <0.4 (18, 19), others did not confirm this association (20, 21). In older subjects, the persistence of high levels of CD8+ lymphocyte activation after one year of virological suppression is associated with an increased risk of AIDS and non-AIDS events, and chronic CD8+ proliferation has been signified as a risk factor for non-Hodgkin’s lymphoma, acute myocardial infarction, and functional impairment (22, 23).

Determining the role of CD4/CD8 ratio or CD8+ count as new prognostic markers would help identify those patients at high risk for morbidity and mortality. This information could be used to classify patients at risk for persistent inflammation and age-related conditions, helping us to intensify health interventions and prevention. This systematic review and meta-analysis aimed to evaluate the independent role of the CD4/CD8 ratio and CD8+ T-cell count as predictors of non-AIDS-related mortality in PLHIV on ART.

## Methods

We performed a systematic literature review and meta-analysis and followed the PRISMA statement to report our findings (24). The study protocol was prospectively registered in PROSPERO (CRD42020170931).

### Search strategy and selection criteria

Studies were eligible if they included PLHIV ≥18 years starting or on current ART with undetectable viral load, as defined by the viral load threshold determined by the primary studies. The studies should evaluate the role of CD4/CD8 ratio or CD8+ T cell count as independent prognostic factors after adjustment for key covariates. We considered age, sex, risk factors for HIV transmission, CD4 nadir, Hepatitis C virus (HCV) or Cytomegalovirus (CMV) coinfection, and type of ART as principal covariates. The adjustment for these covariates was not an inclusion criterion but was considered in the risk of bias assessment. The primary outcome was non-AIDS mortality or all-cause mortality at the longest follow-up as provided by primary studies. We set viral suppression as an inclusion criterion to reduce AIDS-related mortality. The secondary composite outcome was non-AIDS events or death, including any definition of non-AIDS events considered by authors if mortality was reported. We included experimental or observational studies, always that had provided adjusted estimates for at least one review outcome. Studies with elite controllers were excluded. A detailed description of the inclusion criteria is included in the supplemental material (**Supplementary 1** **).**

We conducted a systematic search of the literature from January 1996 to January 2023, without language restriction, in Medline (Ovid), Embase (Elsevier), Cochrane Central Register of Controlled Trials (CENTRAL), and Web of Science databases. This period was established to avoid publications before the implementation of highly active antiretroviral therapy. We used controlled vocabulary (such as MeSH terms) and keywords related to the topics “HIV”, “ CD4/CD8 ratio or CD8” and “mortality/death”. The search strategy is described in **Supplementary 1**.

### Study selection, data extraction, and risk of bias assessment

We used Covidence tool (25), for implementing the study selection process. Every study was independently screened by two reviewers at title/abstract and full-text stages. In case of discrepancy, the final decision was solved by a third one. We extracted the data from each included study with a predesigned Microsoft Excel form based on the CHARMS-PF checklist (26), and we piloted the form in three studies for usability. We classified the studies by CD4/CD8 ratio measurement as a categorical or continuous variable. We reached out to several corresponding authors to acquire additional data needed to reconcile differences and establish reference values. We assessed the risk of bias in the included studies with the QUIPS tool, which is designed for prognostic factor studies (26). The tool domains are described in **Supplementary 2**. Two authors assessed the risk of bias in each included study independently. Discrepancies were resolved by discussion and mutual agreement. The quality of evidence was evaluated with GRADE framework, adapted to prognostic studies as previously reported in the literature (27). The items to consider were related to the phase of investigation: with a high level of evidence assigned to cohort studies; study limitations and bias; inconsistency of results: related to the heterogeneity of findings; indirectness: assessing optimal population, prognostic factors and outcomes, and imprecision: to determine the certainty and interpretation of the results.

### Data analysis

We conducted a meta-analysis to assess the risk of “non-AIDS mortality” and “all-cause mortality” in patients with low versus high CD4/CD8 ratios, using the high ratio as the reference category. A secondary analysis extended this to composite outcomes, including “AIDS, non-AIDS events, and all-cause mortality.” Due to the lack of studies reporting the CD4/CD8 ratio as a continuous variable, and the high heterogeneity in ratio measurement, we limited the statistical analysis to studies reporting the ratio as a categorical variable. Due to varying CD4/CD8 ratio cut-offs across studies (ranging from 0.3 to 1), we standardized our approach for better comparability. We focused on studies using clinically relevant cut-offs below 0.5, as supported by prior research linking low ratios to immunosenescence and increased morbidity and mortality (9, 12, 15, 16, 18). For instance, in studies with a 0.3 cut-off, subjects with ratios below 0.3 were categorized as “low ratio,” and those with ratios of 0.3 or higher as “high ratio”. For CD8+ T-cell counts, the cut-offs used across the selected studies were too variable to meta-analyse these results. A detailed description of the data adjustment to these cut-offs is included in **Supplementary 3**. Six studies (28–33) were excluded from the meta-analysis due to deviations from our study protocol but were accounted for in the systematic review based on follow-up after ART initiation and reported outcomes. Reasons for exclusion are reported in **Supplementary 4**.

We computed the effect measures, including Odds Ratio and Hazard Ratios, and their standard errors using each subgroup’s data, and performed a standard random-effects pairwise meta-analysis model to provide a pooled prognostic effect estimate for the primary and secondary outcomes. The conversion of the association measures into OR was discarded given the variability obtained in the results and the risk of bias. Heterogeneity was assessed with the I^2^ statistic and prediction intervals (34). The protocol also included publication bias assessment for each meta-analysis with 10 or more studies by funnel plot representation and Peter’s test (10% level), but due to the scarce number of studies included in the meta-analysis, this evaluation was not possible. All statistical analyses were performed using Stata (StataCorp. 2021. *Stata Statistical Software: Release 17*. College Station, TX: StataCorp LLC).

### Risk of Bias and quality of evidence of the included studies

**Table 1** summarizes the risk of bias in each study according to the QUIPS tool domains. A detailed description is presented in **Supplementary 5**. Items assessing study attrition showed the highest risk of bias. The overall risk of bias in the included studies was high (n=7; 35%) (28, 30, 31, 36, 37, 40, 43), moderate (n=11: 55%) (9, 18, 19, 21, 29, 32, 33, 35, 38, 39, 41), or low (n=2; 10%) (20, 42). Nearly all included studies (n=18; 90%) (9, 18, 19, 21, 28–33, 35–41, 43) lacked a description of the lost and censored participants, their characteristics, and the methodology for statistical imputation of the missing values. Time definition, including the timing of CD4/CD8 ratio measurement (n=7; 35%) (19, 28, 33, 36, 38, 39, 41), as well as the definition of the time to the event, were penalized in most of the studies (n=16; 80%) (9, 18, 19, 21, 28–33, 36, 37, 39, 41–43). Eight studies (40%) (19, 21, 28, 33, 36, 38, 39, 41) reported CD4/CD8 ratio measurement at the baseline evaluation of the cohorts, with subsequent updating of the data during follow-up, but without specifying the frequency of determination. Age and sex were considered in the adjusted analysis in more than half of the included studies (n=17; 85%) (9, 18, 20, 21, 29–33, 35–39, 41–43). The CD4 T-cell nadir or the baseline values reported in the cohorts were included as adjustment covariates in 8 studies (40%) (9, 18, 20, 21, 29, 39, 41, 42), and current or updated CD4 counts during follow-up were also considered as adjustment covariates by some authors (n=8; 40%) (20, 21, 29, 30, 32, 33, 39, 42). Nine studies (45%) (20, 21, 31–33, 35, 39, 41, 42) considered risk factors for HIV infection. Type of ART (n=5) (20, 29, 31, 35, 41), and HCV coinfection (n=5) (19, 28, 31, 33, 42) were each reported in 25% of the studies. For the studies included in the meta-analysis, again age, sex, and risk group were the most frequent adjustment variables, followed by nadir or baseline CD4 T-cell counts.

**Table 1:** Risk of bias assessment for primary studies.

Outcome	Study	Risk of bias with the QUIPS tool						
		Study Participation	Study attrition	Prognostic factor measurement	Outcome measurement	Adjustment for other prognostic factors	Statistical analysis and reporting	Overall RoB
Non-AIDS mortality	Serrano-Villar (18)	LOW	HIGH	LOW	LOW	LOW	MODERATE	MODERATE
	Trickey (21)	LOW	HIGH	LOW	LOW	LOW	LOW	MODERATE
	Martínez-Sanz (35)	LOW	HIGH	LOW	LOW	LOW	MODERATE	MODERATE
All-cause mortality	Serrano-Villar (9)	LOW	HIGH	LOW	LOW	LOW	MODERATE	MODERATE
	Helleberg (32)	LOW	HIGH	LOW	LOW	LOW	MODERATE	MODERATE
	Cervero (36)	MODERATE	HIGH	LOW	LOW	HIGH	HIGH	HIGH
	Lee (37)	MODERATE	HIGH	LOW	LOW	MODERATE	MODERATE	HIGH
	Duffau (38)	LOW	HIGH	LOW	LOW	LOW	MODERATE	MODERATE
	Castilho (29)	LOW	HIGH	LOW	LOW	LOW	MODERATE	MODERATE
	Boettiger (39)	LOW	HIGH	LOW	LOW	LOW	MODERATE	MODERATE
	Liu (31)	MODERATE	HIGH	LOW	MODERATE	MODERATE	MODERATE	HIGH
	Klugman (30)	MODERATE	HIGH	LOW	MODERATE	LOW	MODERATE	HIGH
	Aksak-Wąs (40)	LOW	HIGH	LOW	LOW	HIGH	MODERATE	HIGH
	Novak (41)	LOW	HIGH	LOW	LOW	LOW	MODERATE	MODERATE
Composite (AIDS, non-AIDS event or mortality)	Collin (28)	LOW	HIGH	MODERATE	LOW	LOW	HIGH	HIGH
	Mussini (42)	LOW	LOW	LOW	LOW	LOW	MODERATE	LOW
	Han (19)	LOW	HIGH	LOW	LOW	MODERATE	MODERATE	MODERATE
	Aldrete (43)	LOW	HIGH	LOW	LOW	HIGH	MODERATE	HIGH
	Domínguez (33)	MODERATE	HIGH	LOW	LOW	LOW	MODERATE	MODERATE
	Serrano-Villar (20)	LOW	LOW	LOW	LOW	LOW	MODERATE	LOW

RoB: Risk of Bias.

Other relevant adjustment variables reported in the primary studies were the CDC/WHO classification or history of AIDS and the time since ART initiation. CMV infection was marginally recorded, being the least analyzed covariate. As six of the seven studies included in the meta-analysis were graded with a moderate overall risk of bias, we did not perform sensitivity analyses. The overall quality of evidence was moderate for the studies evaluating the CD4/CD8 ratio as a prognostic factor for non-AIDS mortality and all-cause mortality included in the meta-analysis. For the studies included in the systematic review assessing the role of the CD4/CD8 ratio or CD8+ T-cell counts as prognostic markers, the overall quality was low, mainly related to the risk of bias and the variability in results across studies. A detailed report of the adapted GRADE framework used is presented in **Supplementary 6**.

## Results

### Study characteristics

**Figure 1:**
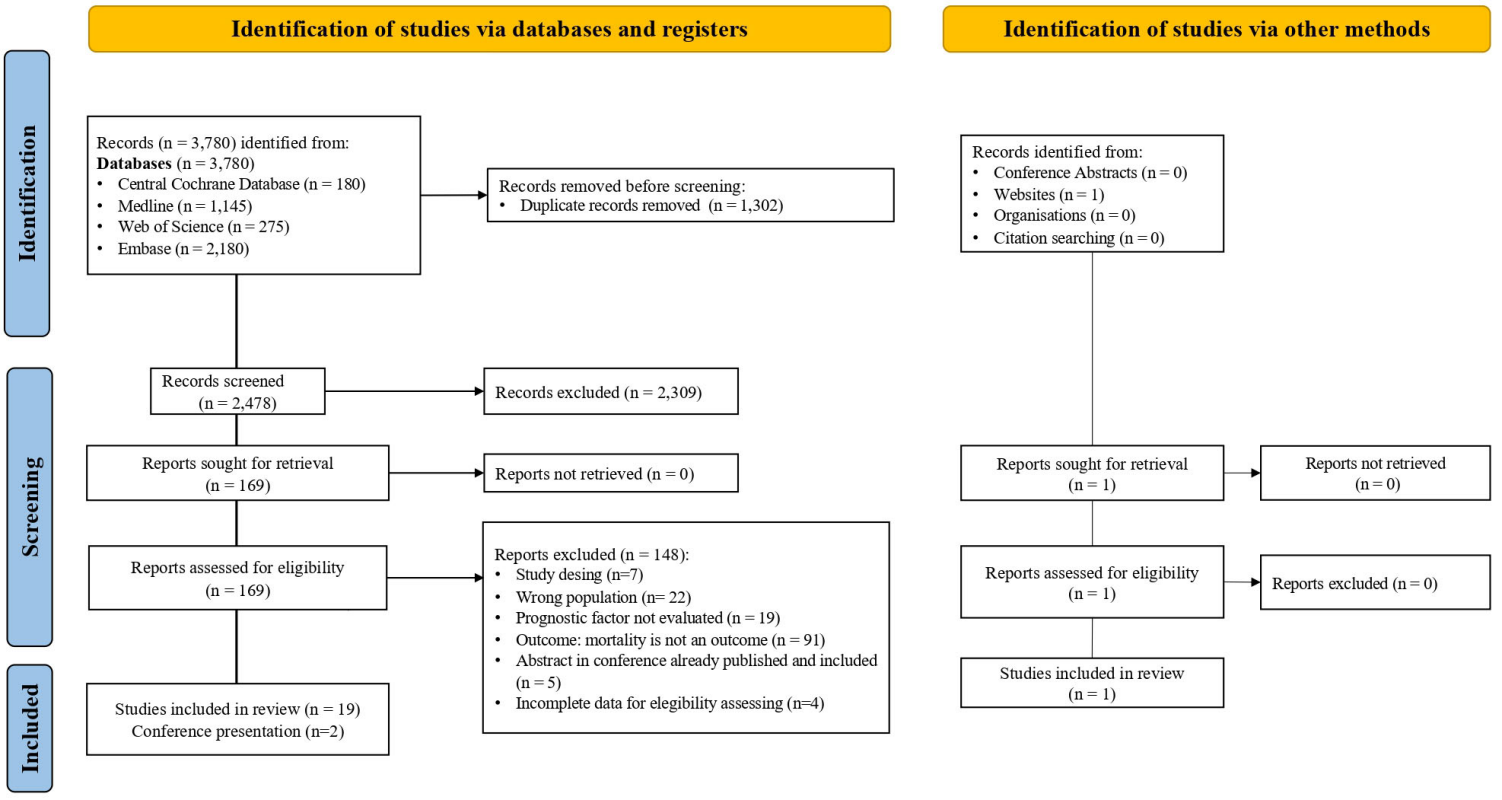
Studies selection flowcharts showing identification, screening, inclusion and exclusion process.

We screened 2,478 studies and identified 169 for eligibility. Studies selection flowchart is represented in **Figure 1**. After a full-text review, we included 20 studies in the systematic review. Most of the included studies (n=18) were hospital-based multicentric prospective cohorts, with two case-control studies (9, 18). The observed follow-up period ranged between 3 and 16 years, with a total number of 184,402 participants and 6,940 reported deaths (number of deaths not reported in all studies). The general characteristics of the primary studies are described in **Table 2**. The threshold defined for virological suppression went from 20 copies/mL to 1,000 copies/mL. Except for the study by Helleberg et al. (32), which reported only the CD8+ counts, all the selected studies measured CD4/CD8 ratio, with six studies reporting CD8+ T-cells as independent values. The main results are detailed in **Table 3**.

**Table 2:** Characteristics of the selected studies.

Study	Country	Study period	Observed follow-up (yr.)	Study design	Sample size	Age (yr.)	Sex (F/M)	HIV acquisition (%)	Baseline viral load (copies/mL)	Outcomes
**Serrano-Villar et al.** (18)	Spain	1999-2012	NA Median cumulative ART exposure 3.9-9.8	Case-control	407	Cases: 46 Controls: 41	80/327	PWID - 53 (48) MSW - 26 (24) MSM - 23 (21)	At least one year of viral suppression	Non-AIDS mortality
**Serrano-Villar et al. (** 9 **)**	USA	NA	NA Median cumulative ART exposure 3-10	Nested case-control	183	44	38/154	NA	<400	All-cause mortality
**Helleberg et al. (** 32 **)**	Denmark	1995-2012	Median 8.6 (4.1-13.7)	Prospective cohort	3882	39	822/3060	PWID - 388 (10) MSW - NA MSM - 2012 (52)	ART naive at baseline On year 1: 71% with VL <40	All-cause mortality
**Mussini et al. (** 42 **)**	Italy	1997-2013	NA (>5) Median time to ratio normalization 10.1	Prospective cohort	3236	39	765/2471	PWID - 669 (21) MSW - 1339 (41) MSM - 1031 (32)	≤80	Composite: Non-AIDS events or death
**Cervero et al. (** 36 **)**	Spain	1985-2014	NA Median cumulative ART exposure 15 (5–17)	Prospective cohort	142	48	45/97	PWID - 60 (42) MSW - 53 (37) MSM - 21 (15)	<20	Non-AIDS events All-cause mortality
**Collin et al.,** (28)	France	2000-2012	Median 7.4 (3.2-12.3)	Prospective cohort	5354	39	177/481	PWID - 177 (27) MSW - 195 (30) MSM - 219 (33)	Only 27% with ≤50	Composite: Non-AIDS severe bacterial infections or death
**Lee et al. (** 37 **)**	Uganda	2005-2013	Median 7	Prospective cohort	535	34	370/165	NA	<400 at Month 6 of ART	All-cause mortality
Trickey et al.,* (21)	Multicentric	1996-2013	10	Prospective cohort	49865	37	13724/36141	PWID - 3638 (7) MSW - NA MSM - NA	<200	Non-AIDS mortality AIDS mortality All-cause mortality
**Duffau et al.,** (38)	France	2011- 2016	3	Prospective cohort	828	51	206/622	PWID - 115 (14) MSW - 281 (34) MSM - 359 (43)	<40	Comorbidity All-cause mortality
Han et al.,* (19)	Thailand	1996-2017	10	Prospective cohort	800	32	267/533	PWID - 6 (0.75) MSW - 433 (54) MSM - 280 (35)	< 50	Composite: Non-AIDS events or death
**Castilho et al.,** (29)	USA	1998-2015	16	Prospective cohort	341 (116 on VS)	49	70/271	PWID - 55 (16) MSW - 103 (30) MSM - 166 (49)	<400 for at least 80% of observation time before event	All-cause mortality
**Aldrete et al.,** (43)	USA	Median follow- up 5.4y (3.4-7.9)	12	Prospective cohort	2422	38	315/2107	NA	<1,000	Composite: AIDS, non-AIDS events or death
Boettiger et al.,* (39)	Brazil	2003-2014	5	Prospective cohort	5381	37	NA	PWID - 170 (3) MSW - 2845 (53) MSM - 1628 (30)	On ART for at least 6 months <65	Cardiovascular disease All-cause mortality
**Liu et al.** (31)	China	2005-2018	12	Prospective cohort	91805	37	17086/74719	PWID - 5771 (6) MSW - 40366 (44) MSM - 40511 (44)	ART naive at baseline	All-cause mortality
**Klugman et al.** (30)	USA	2004-2017	15	Prospective cohort	2969 (88 HIV+)	57	35/53	NA	43% had detectable VL nearest to lung cancer diagnosis	All-cause mortality
**Aksak-Wąs et al.** (40)	Poland	2010-2020	10	Prospective cohort	1727	35	259/1468	NA	VL<50 for at least 6 months	All-cause mortality
**Domínguez et al.** (33)	Spain	2004-2018	12	Prospective cohort	10018	36	NA	PWID - 707 (7) MSW - 1560 (16) WSM - 1334 (13) MSM - 6071 (61)	ART naive at baseline	Composite: AIDS or non-AIDS events or all-cause mortality
Novak et al.,* (41)	USA	2000-2019	Median 7.4 (4.1-12.2)	Prospective cohort	2480	<40: 1145 40-49: 835 ≥50: 500	457/2023	PWID - 155 (6) MSW - 625 (25) MSM - 1561 (63)	<200 At least one year on ART	Non-AIDS events All-cause mortality
Serrano-Villar et al.,* (20)	USA	1998-2011	7	Prospective cohort	5133	38	959/4174	PWID - 407 (8) MSW - NA MSM - NA	<200 at year 2 of ART	Composite: AIDS, non-AIDS events or death
Martínez-Sanz et al.,* (35)	Spain	2004-2014	7	Prospective cohort	4625	37	773/3852	PWID - 1477 (32) Heterosexual - 391 (8) MSM - 2609 (56)	<50 at year 2 of ART	Composite: Non-AIDS events or death Non-AIDS mortality

*Studies included in the meta-analysis. Age is reported in mean/median years. (yr.): years. NA: not available. ART, antiretroviral treatment; PWID, people who inject drugs; MSW, men who have sex with women; MSM, men who have sex with men; VL, viral load; VS, viral suppression.

**Table 3:** Main results of selected studies.

Study	Prognostic Factor	Prognostic factor measurement	Deaths	Outcome measurement	Study reported effect	Adjusted reported effect	Covariates evaluated	Direction of association
Serrano-Villar et al.,* (18)	CD4/CD8	6 months before the event	29	Not defined	OR;95% CI	**CD4/CD8 ratio <0.4:** 4.5 (1.7–11.8)	Age, sex, CD4 nadir, first ART date, years on ART	Ratio <0.4 associated with high risk of non-AIDS mortality
**Mussini et al.,** (42)	CD4/CD8	Every 4 months	13	Not defined	RR;95% CI	**Reference CD4/CD8 ratio >0.45** 0.30-0.45: 0.95 (0.69–1.31) <0.30: 1.51 (1.09–2.09)	Age, HIV transmission, years from HIV diagnosis, time to VS, baseline CD4, current CD4, HCV, CDC stage	Ratio <0.3 associated with high risk of non-AIDS events and mortality
Trickey et al.,* (21)	CD4/CD8 CD8	Baseline and time updated (not specified)	1834	Not defined	HR;95% CI	**CD4/CD8 ratio** **Reference >0.4** All-cause mortality ≤0.4: 1.08 (0.98-1.20) AIDS mortality ≤0.4: 1.42 (1.08-1.86) Non-AIDS mortality ≤0.4: 1.03 (0.90-1.17) **CD8 count. Reference (761–1138):** All-cause mortality 0-760: 1.05 (0.93–1.18) >1138: 1.13 (1.01–1.26) Non-AIDS mortality 0-760: 1.09 (0.93–1.27) >1138: 1.10 (0.95–1.27)	Age, sex, CDC stage, current CD4 (>350), baseline viral load, years on ART, intravenous drug use	Ratio ≤0.4 not associated with non-AIDS mortality. Weak evidence that non-AIDS mortality was higher in the upper tertiles of CD8 counts
Han et al.,* (19)	CD4/CD8	At first viral suppression and current (not specified)	15	Not defined	HR;95% CI	**Reference CD4/CD8 ratio >0.45** <0.3: 3.02 (1.27–7.21) 0.30-0.45: 2.03 (1.03–3.98)	Baseline CDC stage, HCV, cancer diagnosis, HIV viral load, DM	Ratio <0.45 associated with high risk of non-AIDS events and mortality
Boettiger et al.,* (39)	CD4/CD8	Baseline and time updated (Not specified)	418	Not defined	HR;95% CI	**Reference CD4/CD8 ratio >0.7** 0.4-0.7: 1.1 (0.8–1.5) <0.4: 2.5 (1.8-3.4) **Estimated OR for CD4/CD8 ratio <0.4 vs ≥0.4** 3.63 (2.93-4.5)	Age, sex, HIV transmission, previous CVD, HBP, DL, DM, smoke, nadir CD4, current CD4, HIV viral load, previous AIDS, first ART date	Ratio <0.4 associated with high risk of all-cause mortality
Novak et al.,* (41)	CD4/CD8	Baseline and most recent before events	124	Not defined	RR;95% CI OR; 95% CI	**Reference CD4/CD8 ratio ≥0.5** RR: 3.66 (2.61-5.12) OR: 4.01 (2.79-5.78)	Age, sex, race, HIV transmission, smoke, type of ART, baseline CD4	Ratio <0.5 associated with high risk of all-cause mortality
Serrano-Villar et al.,* (20)	CD4/CD8 CD8	After 2 years of ART	32	Years 3-7 on ART	OR;95% CI	**CD4/CD8 ratio** **Reference >0.3** <0.15: 1.34 (0.76,2.39) 0.15-0.3: 0.73 (0.57,0.93) **CD8 count. Reference (500–1500)** <500: 1.21 (0.97, 1.51) ≥1500: 1.91 (1.47, 2.49)	Age, sex, race, current CD4, type of ART, Intravenous drug use, baseline viral load, previous events	Ratio ≤0.3 not associated with a high risk of AIDS, non-AIDS events, or mortality CD8 ≥1500 cells/μL associated with high risk
Martínez-Sanz et al.,* (35)	CD4/CD8 CD8	After 2 years of ART	47	Years 3-7 on ART	OR;95% CI	**CD4/CD8 ratio. Reference ≥0.3** Non-AIDS events or mortality <0.3: 1.62 (1.03-2.58) Non-AIDS mortality OR 1.40 (0.50-3.91) **CD8 count ≥800** OR 1.12 (95% CI 0.71-1.76) **CD8 count ≥1000** OR 1.25 (95% CI 0.81-1.93) **CD8 count ≥1500** OR 1.78 (95% CI 1.03-3.08)	Age, sex, HIV transmission, educational level, country of origin, previous AIDS, nadir CD4, baseline HIV viral load, type of ART, inclusion date	Ratio <0.3 associated with high risk of non-AIDS events CD8 ≥1500 associated with high risk
**Serrano-Villar et al.,** (9)	CD4/CD8 CD8	18 months before event	62	Not defined	Beta coefficient	**CD4/CD8 Ratio** log transformed Beta -1.38 St error 0.55 **CD8 count** Beta 0.28 St error 0.33	Age, sex, nadir CD4, time on VS	Ratio associated with high risk. For each 10% increase in the CD4/CD8 ratio there was a 15% decrease in risk of all-cause mortality CD8 count not associated
**Helleberg et al.,** (32)	CD8	Baseline (pre ART)/1year/10 years	824	0, 1 and 10 years on ART	MRR (95% CI)	**CD8 count. Reference (500–1499)** 1y mortality CD8 <500: 1.81 (1.30-2.50) CD8 1500-2000: 1.06 (0.70-1.59) CD8 >2000: 1.61 (0.94-2.75) 10y mortality CD8 <500: 0.93 (0.39-2.23) CD8 1500-2000: 2.03 (1.09-3.80) CD8 >2000: 1.99 (0.98-4.03)	Age, sex, HIV transmission, current CD4, year of HIV diagnosis	CD8<500 cells/μL on the first year of ART associated with high risk of all-cause mortality. CD8>1500 cells/μL after 10y on ART associated with high risk
**Cervero et al.,** (36)	CD4/CD8	Baseline and time updated (not specified)	10	Not defined	OR;95% CI	**CD4/CD8 ratio <0**.**7** OR 5.96 (IC 95% 0.73-48.40)	Age	Ratio <0.7 associated with high risk of all-cause mortality
**Collin et al.,** (28)	CD4/CD8	Baseline and time updated (not specified)	NA	Not defined	HR;95% CI	**CD4/CD8 ratio Reference ≥ 1** 0.8-1.0: 1.27 (0.85-1.90) 0.5-0.8: 1.93 (1.40-2.67) 0.3-0.5: 2.18 (1.56-3.05) <0.3: 3.84 (2.77-5.32)	CDC stage, HCV, cancer, HIV viral load, DM	Ratio <0.8 associated with high risk of severe non-AIDS infections or death
**Lee et al.,** (37)	CD4/CD8	Pre ART and at month 6 of viral suppression	25	Month 6 on ART	HR;95% CI	**CD4/CD8 ratio. Reference ≥0.4** <0.4: 0.47 (0.12-1.8)	Age, sex, pre-ART CD4, BMI, baseline HIV viral load	Ratio <0.4 not associated with high risk of all-cause mortality
**Duffau et al.,** (38)	CD4/CD8	Baseline	24	3 years	HR;95% CI	**CD4+/CD8 ratio. Reference ≥1** 0.7 (0.2-1.9)	Age, sex, CDC stage, baseline CD4	Ratio <1 not associated with high risk of all-cause mortality
**Castilho et al.,** (29)	CD4/CD8	365–60 days before event	129	Not defined	HR;95% CI	**CD4/CD8 ratio (per 0.1 increase)** 0.89 (0.76–1.04)	Age, sex, type of ART, nadir CD4 and before event	Low ratio not associated with high risk of all-cause mortality
**Aldrete et al.,** (43)	CD4/CD8	Year 2 of ART	9	After 2 years of ART	HR;95% CI	**Two stage model** **Estimated CD4/CD8 Intercept** Per 0.1 ratio increase: 0.97 (0.9–1.01) **Estimated CD4/CD8 Slope** 0.1 higher rate per year: 0.74 (0.63–0.86)	Age, cohort of origin	CD4/CD8 ratio slope >0.15 per year had a composite outcome (AIDS, non-AIDS or death) of 14.2% (11.3%-17.7%) versus 28.8% (24.4%-33.9%) for ≤ 0.15
**Liu et al.,** (31)	CD4/CD8 CD8	Baseline (pre-ART)	3134	Not defined	HR;95% CI	**CD8 count. Reference ≥1000** <500: 1.15 (1.0 3-1.28) 500-999: 0.97 (0.87-1.07)	Age, sex, HIV transmission, WHO clinical stage, HCV, baseline CD4, type of ART	Lower baseline CD8 count associated with high risk of all-cause mortality. Cumulative probability of death higher with baseline CD4/CD8 ratio ≤0.19
**Klugman et al.,** (30)	CD4/CD8	Prior to cancer diagnosis	61	Not defined	HR;95% CI	**CD4/CD8 ratio. Reference ≥0.43** <0.43: 1.37 (0.71-2.62)	Age, sex, cancer stage, current CD4, HIV viral load	Ratio <0.43 not associated with high risk for all-cause mortality
**Aksak-Wąs et al.,** (40)	CD4/CD8	Baseline (pre-ART) Time updated at pre-defined immune recovery	NA	Not defined	HR;95% CI	NA	NA	Ratio ≤1 and ≤0.8 associated with high risk of all-cause mortality
**Domínguez et al.,** (33)	CD4/CD8	Baseline and time updated (not specified)	298	Not defined	HR;95% CI	**Non-late presenters** **CD4/CD8 Ratio. Reference >0.4** ≤0.4: 1.39 (0.96-2.02) **Late presenters** **CD4/CD8 ratio. Reference >0.4** ≤0.4: 1.62 (1.10-2.40)	Age, sex, current CD4, HIV transmission, HCV, HBV, educational level, country of origin	Ratio ≤0.4 associated with high risk of composite outcome. Higher risk in late presenters

*Studies included in the meta-analysis; CD4 and CD8 count reported as cells/mm3. OR; Odds Ratio; HR, Hazard Ratio; RR, Risk Relative; NA, not available; ART, antiretroviral treatment; BMI, body mass index; VS, viral suppression; CDC, Centre for Diseases Control; WHO, World Health Organisation; HCV, hepatitis C virus; HIV, human immunodeficiency virus; AIDS, acute immunodeficiency syndrome; DM, diabetes mellitus; DL, dyslipidemia; HBP, high blood pressure.

### Mortality risk (non-AIDS mortality or all-cause mortality)

#### CD4/CD8 ratio

**Figure 2:**
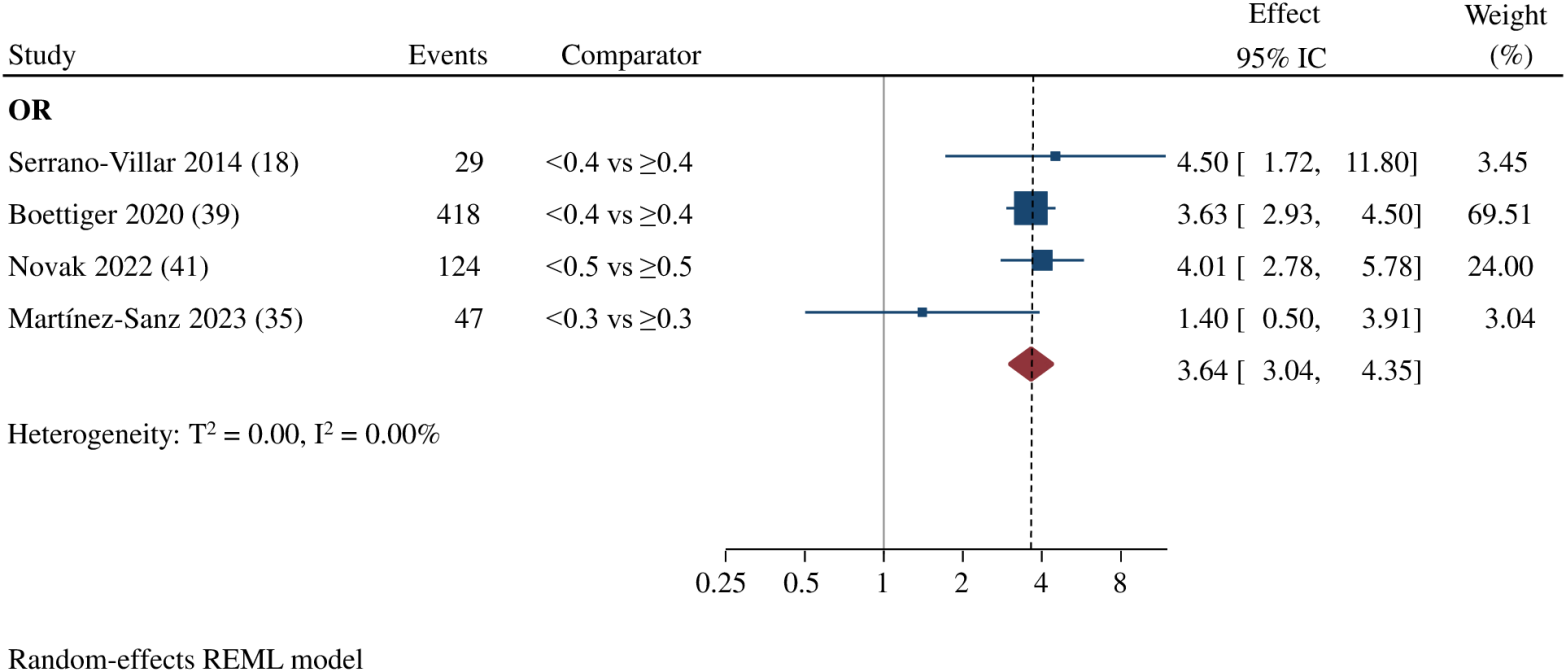
Estimated effect of CD4 /CD8 ratio on the risk on non-AIDS mortality or all-cause mortality. Random effect of meta-analysis comparing low vs. high CD4/CD8 ratios. (OR, Odds Ratio). Events include the total number of deaths reported.

Among twenty studies included in the systematic review, three (18, 21, 35) evaluated “non-AIDS mortality”, and twelve (9, 19, 21, 30–32, 36–41) reported “all-cause mortality” as independent outcomes, for a total follow-up between 3 and 16 years (**Table 2**). Seven of these studies (9, 18, 31, 36, 39–41) found an association between the lower CD4/CD8 ratio categories and increased mortality risk, with variable cut-off points reported, including 0.4, 0.5, 0.7, 0.8, and 1. Most of these studies reported the last ratio measurement before the development of the event or updated during follow-up. After considering the studies with our pre-specified cut-off values, the meta-analysis of non-AIDS or all-cause mortality (18, 35, 39, 41) showed an increased risk of mortality in subjects with a CD4/CD8 ratio below 0.5 (OR 3.64; 95% CI 3.04-4.35; 4 studies; n= 12,893; Follow-up: range 4.1 to 12.2 years; I^2 =^ 0.00%; 95% Prediction interval: 2.46 to 5.38) compared to those above 0.5, with a reference threshold between 0.3 and 0.5 (**Figure 2** **).**

#### CD8+ T-cell count

Analysis of CD8+ T-cell count as an independent mortality predictor was not feasible, due to high variability in cut-off points, reference values, and timing of CD8+ measurement concerning ART initiation and the clinical event (**Table 3** **).** The systematic review suggests a double trend. First, lower baseline CD8+ levels appear to be associated with a higher risk of overall mortality. Two studies found an association with higher mortality risk in subjects with CD8+ count <500 cells/uL before ART initiation and in the first year of treatment (31, 32). Furthermore, four studies reported an increased risk of mortality or clinical events in virally suppressed patients with CD8+ counts in the higher categories (with cut-offs ranging from 1138 to 1,500 cells/uL) (20, 21, 32, 35), suggesting that the persistence of high CD8+ T-cell counts represent a negative prognostic factor in PLHIV on ART.

#### Composite outcomes: AIDS clinical outcomes, non-AIDS clinical outcomes, or mortality

**Figure 3:**
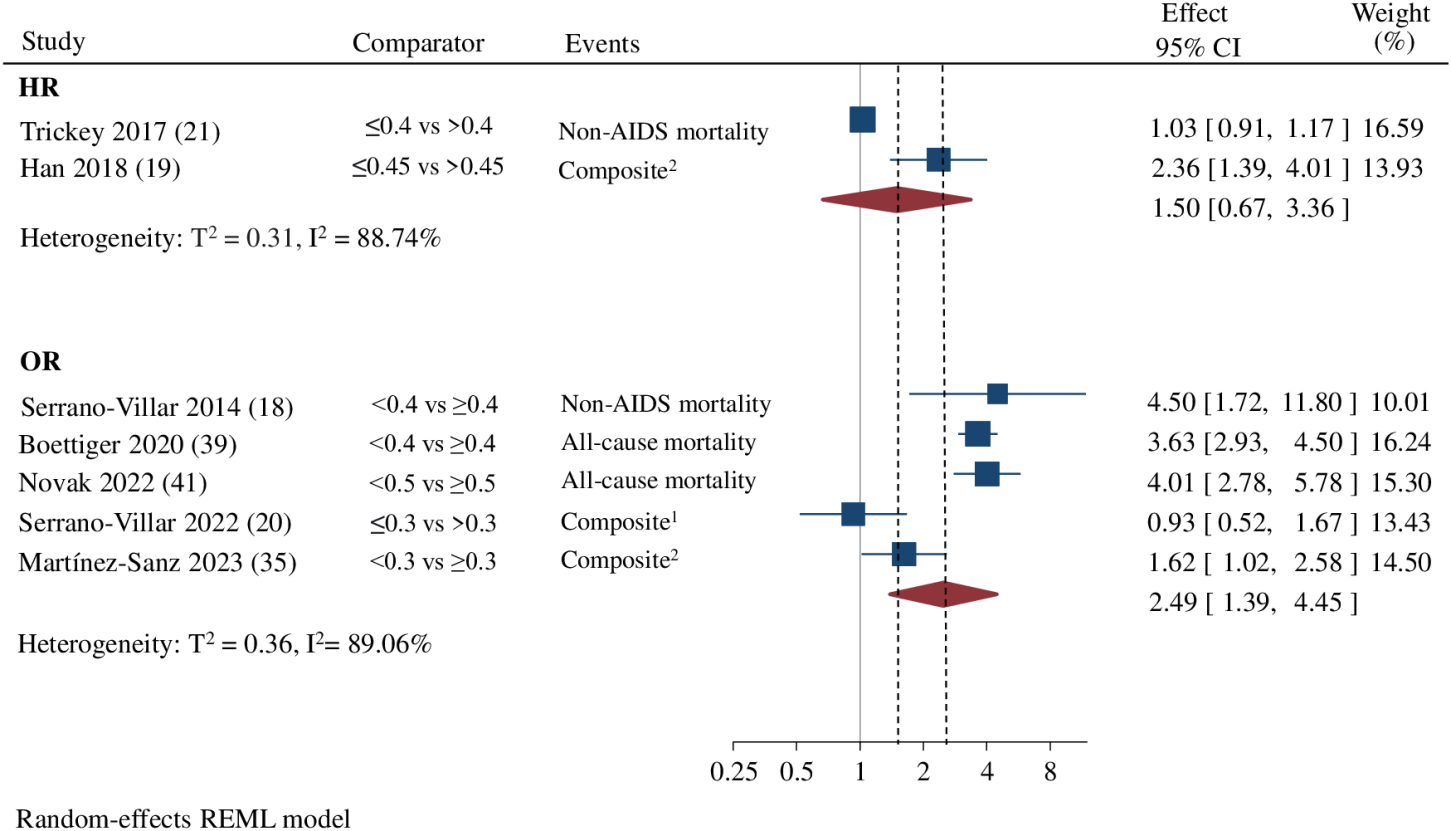
Estimated effect of CD4/CD8 ratio on the risk of clinical events or mortality. Random effects meta-analysis by association measurement (HR, Hazard Ratio/OR, Odss Ratio) comparing low vs. high CD4/CD8 ratios. ^1^ composite including AIDS, non-AIDS events, and all-cause mortality. ^2^ composite outcomes including non-AIDS events and non-AIDS mortality.

Seven studies looked at the risk for composite outcomes including AIDS, non-AIDS events, and mortality (19, 20, 28, 33, 35, 42, 43), with five authors finding an association with an increased risk of events for CD4/CD8 ratio values below 0.3 and 0.8 (19, 28, 33, 35, 42). Due to differences in effect measurement, statistical methodology, and baseline characteristics of the cohorts, it was not possible to conduct a meta-analysis to assess the risk of composite events independently. Therefore, primary studies with composite events were evaluated together with mortality outcomes in a second analysis. The meta-analysis including five studies reporting OR as the effect measure (18, 20, 35, 39, 41) showed an increased risk of AIDS, non-AIDS clinical events, or mortality in subjects with lower CD4/CD8 ratio categories (OR:2.49; 95% CI 1.39-4.45; 5 studies; n= 18,026 patients; Follow-up: 4.1-12.2 years; I^2 =^ 89.06%; 95% Prediction interval: 0.30 to 20.94), compared to those with higher values, with cut-offs and reference thresholds ranging between 0.3 and 0.5. For two studies reporting HR (19, 21), we did not detect a significant effect of a CD4/CD8 ratio below 0.4 (HR 1.50, 95% CI 0.67-3.36; 2 studies; n= 50,665 patients; Follow-up: 10 years; I^2 =^ 88.74%; 95% Prediction interval not calculable) compared to those with values above 0.45, with cut-offs and reference thresholds ranging between 0.4 and 0.45. (**Figure 3**). Statistical heterogeneity was high for both analyses.

Sixteen studies evaluated a spectrum of non-AIDS events (**Supplementary 7**). The most prevalent reported were cardiovascular and cerebrovascular disease (acute myocardial infarction, coronary heart disease, and stroke); chronic renal and liver disease; and non-AIDS-defining cancers. Four studies also considered AIDS events (20, 21, 33, 43), two of them as part of the composite outcome definition. All these analyses were performed or stratified by patients under ART-mediated viral suppression. In the study by Aldrete et al. (43), AIDS events accounted for 27.3%, determined by a poor CD4 or CD4/CD8 ratio recovery in the first years of ART. The study by Serrano-Villar et al. (20) reported a 7% of AIDS events.

## Discussion

This is, to our knowledge, the first systematic review evaluating the CD4/CD8 ratio as a predictor of mortality in PLHIV. Considering assessing the predictive value of the CD4/CD8 ratio, our primary focus was on mortality, a robust event for which most previous studies lacked sufficient statistical power to produce precise estimates. The findings from our study provide evidence to suggest that a low CD4/CD8 ratio, as defined by values below 0.3, 0.4, or 0.5 across different studies, serves as an adverse prognostic indicator for non-AIDS and all-cause mortality. Based on the evaluated evidence, PLHIV on ART who exhibit low CD4/CD8 ratios may face more than a threefold increase in mortality risk compared to those with higher ratios. When considering composite outcomes including comorbidities and mortality, we also found an association between lower CD4/CD8 ratios and high risk of AIDS, non-AIDS events, and all-cause mortality.

Persistent immune imbalance in PLHIV and its possible related outcomes have been evaluated by different authors. Recently data have confirmed an increased risk of different types of cancer, including AIDS and non-AIDS cancers in patients with lower ratios (0.30 vs. 0.80). These observations were made up to two years before diagnosis, suggesting the potential utility of the CD4/CD8 ratio as a clinical biomarker (44). Concerning cardiovascular disease, a low CD4/CD8 ratio has been associated with a higher prevalence of coronary atherosclerosis in young men living with HIV and virological suppression, in association with other classical cardiovascular risk factors (45). Previous studies have also unveiled a correlation between heightened CD8+ lymphocyte activation, persistently high CD8+ count, and an increased risk of both AIDS and non-AIDS events (22, 23, 46, 47). The study by Badejo et al. (48) explored the relationship between CD8+ counts and myocardial infarction risk. A higher risk of acute myocardial infarction (AMI) was observed in patients with CD4+ counts less than 200 and low CD8+ counts, while those with CD4+ counts over 200 cells/uL had a higher risk when CD8+ values were elevated. These findings align with results from several studies in our review. It suggests that imbalances in CD8^+^ levels may exert varying impacts, largely depending on the timing of the assessment. This differential effect is discernible both at baseline, characterized by lymphocyte loss and immunosuppression, and during chronic ART where excessive immune activation and persistently high counts are observed.

In this context, the definition of a cut-off point with the greatest predictive capacity is essential, to confirm a prognostic impact of the CD4/CD8 ratio or CD8+ count, homogenize conclusions of future studies, and facilitate the implementation of the ratio as a risk marker in clinical practice. Although a CD4/CD8 ratio <1 is considered indicative of immune dysfunction in the general population (49), initial studies in PLHIV on ART demonstrated the predictive capacity of lower cut-off points (0.3-0.4) (18, 42). Values close to this cut-off have proven useful in identifying patients with increased immune dysfunction despite a high CD4 count. In the most recent study included in our review (35), several cut-off points were specifically evaluated, and the value of 0.3 was found to discriminate the risk of non-AIDS events.

Given the methodological heterogeneity in the studies related to the impact of CD8+ count, they were unsuitable for inclusion in a meta-analysis. Regarding the most discriminative threshold for CD8+ count, three of the reviewed studies, reporting similar cut-off points and reference values showed that in people on ART, CD8+ counts >1,500 cells/uL, were associated with a significant increase in the risk of clinical events and mortality (32) (20) (35). Also, the study by Trickey et al. (21), including patients with ≥350 CD4, found a moderate risk of all-cause mortality among subjects with high CD8+ levels (>1,138 cell/uL). CD8+ count seems to be determinant both in the initial immune response to HIV infection as well as in the maintained immune activation during ART, but future studies are needed to explore the applicability of these cut-off values on specific clinical events.

Our study has several limitations. The main issue was the heterogeneity in CD4/CD8 ratio and CD8+ measurement between studies. The differences in the established cut-off points, its report as a continuous or categorical variable with varying intervals, and the variability of the reference values (ranging from ≥0.3 to ≥1 for the CD4/CD8 ratio) have been the main drawbacks, restricting the number of studies included in the meta-analysis. Another key factor is the timing of ratio measurement concerning ART initiation and the subsequent outcomes, with some studies clearly defining these times, while others employ a follow-up strategy with flexible time intervals. This is particularly important given the stability of the CD4/CD8 ratio as a parameter, especially for assessing non-AIDS events in patients already receiving treatment (50). Additionally, there was variation in the laboratory threshold for viral suppression, which we set as an inclusion criterion to minimize AIDS mortality. The evolution of ART regimens over time contributes to the heterogeneity, with potential implications for morbidity and mortality. Covariates, such as CMV seropositivity, HCV co-infection, and risk factors for comorbidities, were inconsistently recorded across the studies. The geographical location of the studies may affect the generalizability of our results, as most were conducted in Western European or North American countries, where treatment coverage and clinical care standards for HIV patients might differ significantly from other regions. Finally, the risk of bias assessment underscores the ongoing need for further research of higher methodological quality. This is particularly relevant concerning study attrition, an area that requires more comprehensive exploration to provide more accurate answers to our critical questions.

In conclusion, our systematic review and meta-analysis highlight the significant role of the CD4/CD8 ratio as a prognostic indicator for mortality and non-AIDS events in PLHIV on ART. However, to enhance the validity and applicability of these findings, future studies should pursue a more uniform design, with predefined, standardized measures and intervals for both the ratio and events, a clear definition of censoring timings, and a particular focus on non-AIDS events and mortality. Leveraging the CD4/CD8 ratio as a mortality biomarker presents an opportunity to define an “immunological threshold”, potentially enabling better patient stratification, surveillance, and more targeted preventive measures for those with lower values. This development could bring about direct improvements in routine clinical practice and serve as a starting line in the search for ratio recovery strategies and future research.

## Data availability statement

The original contributions presented in the study are included in the article/**Supplementary Material**. Further inquiries can be directed to the corresponding author.

## Author contributions

RR: Conceptualization, Data curation, Formal analysis, Investigation, Writing – original draft, Writing – review & editing. JM-S: Data curation, Investigation, Writing – review & editing. SH: Data curation, Investigation, Writing – review & editing. LR-R: Data curation, Investigation, Writing – review & editing. AD: Data curation, Investigation, Writing – review & editing. TS: Data curation, Investigation, Writing – review & editing. NÁ-D: Data curation, Methodology, Writing – review & editing. AC-P: Data curation, Methodology, Writing – review & editing. AM: Data curation, Formal analysis, Methodology, Writing – review & editing. JL-A: Conceptualization, Methodology, Supervision, Writing – review & editing. JP-M: Methodology, Supervision, Writing – review & editing. SM: Supervision, Writing – review & editing. SS-V: Conceptualization, Data curation, Formal analysis, Investigation, Methodology, Supervision, Writing – original draft, Writing – review & editing.
