# Corrigendum: CD4/CD8 ratio and CD8+ T-cell count as prognostic markers for non-AIDS mortality in people living with HIV. A systematic review and meta-analysis

**DOI:** 10.3389/fimmu.2024.1383117

**Published:** 2024-02-20

**Authors:** Raquel Ron, Javier Martínez-Sanz, Sabina Herrera, Luis Ramos-Ruperto, Alejandro Díez-Vidal, Talía Sainz, Noelia Álvarez-Díaz, Andrea Correa-Pérez, Alfonso Muriel, Jesús López-Alcalde, José A. Pérez-Molina, Santiago Moreno, Sergio Serrano-Villar

**Affiliations:** ^1^ Department of Infectious Diseases, Hospital Universitario Ramón y Cajal and Instituto Ramón y Cajal de Investigación Sanitaria (IRYCIS), Madrid, Spain; ^2^ Universidad de Alcalá (UAH), Madrid, Spain; ^3^ CIBER de Enfermedades Infecciosas (CIBERINFEC), Instituto de Salud Carlos III, Madrid, Spain; ^4^ Infectious Diseases Department, Hospital Clinic, Barcelona, Spain; ^5^ HIV Unit, Internal Medicine Department, Hospital Universitario La Paz, Madrid, Spain; ^6^ Pediatric Tropical and Infectious Diseases, Hospital la Paz and La Paz Research Institute (IdiPAZ), Center for Biomedical Research in Infectious Diseases (CIBERINFEC), Universidad Autónoma de Madrid, UAM, Madrid, Spain; ^7^ Medical Library, Hospital Universitario Ramón y Cajal, Instituto Ramón y Cajal de Investigación Sanitaria (IRYCIS), Madrid, Spain; ^8^ Faculty of Medicine, Universidad Francisco de Vitoria (UFV), Madrid, Spain; ^9^ Pharmacy and Medical Devices Department. Hospital Central de la Defensa Gómez Ulla, Madrid, Spain; ^10^ Clinical Biostatistics Unit, Hospital Universitario Ramón y Cajal, Instituto Ramón y Cajal de Investigación Sanitaria (IRYCIS), Centro de Investigación Biomédica en Red. Epidemiología y Salud Pública (CIBERESP), Madrid, Spain; ^11^ Institute for Complementary and Integrative Medicine, University Hospital Zurich and University of Zurich, Zurich, Switzerland

**Keywords:** HIV, CD4/CD8 ratio, mortality, comorbidities, non-AIDS events

In the published article, there was an error in affiliations 1-10. The following is the incorrect author list and affiliations:

Raquel Ron^1,2^*, Javier Martínez-Sanz^1,2^, Sabina Herrera^3^, Luis Ramos-Ruperto^4^, Alejandro Díez^4^, Talía Sainz^5^, Noelia Álvarez-Díaz^6^, Andrea Correa-Pérez^7,8,9^, Alfonso Muriel^9^, Jesús López-Alcalde^7,9,10^, José A. Pérez-Molina^1,2^, Santiago Moreno^1,2^ and Sergio Serrano-Villar^1,2^

1. Department of Infectious Diseases, Hospital Universitario Ramón y Cajal and IRICYS, Universidad de Alcalá (UAH), Madrid, Spain

2. CIBER de Enfermedades Infecciosas (CIBERINFEC), Instituto de Salud Carlos III, Madrid, Spain

3. Infectious Diseases Department, Hospital Clinic, Barcelona, Spain

4. HIV Unit, Internal Medicine Department, Hospital Universitario La Paz, Madrid, Spain

5. Pediatric Tropical and Infectious Diseases, Hospital La Paz and La Paz Research Institute (IdiPAZ), Center for Biomedical Research in Infectious Diseases (CIBERINFEC), Universidad Autónoma de Madrid, UAM, Madrid, Spain

6. Medical Library, Hospital Universitario Ramón y Cajal, IRYCIS, Madrid, Spain

7. Faculty of Medicine, Universidad Francisco de Vitoria (UFV), Madrid, Spain

8. Pharmacy and Medical Devices Department. Hospital Central de la Defensa Gómez Ulla, Madrid, Spain

9. Clinical Biostatistics Unit, Hospital Universitario Ramón y Cajal, IRYCIS, CIBERESP, Universidad de Alcalá, Madrid, Spain

10Institute for Complementary and Integrative Medicine, University Hospital Zurich and University of Zurich,

Zurich, Switzerland

And the following is the correct author list and affiliations:

Raquel Ron^1,2,3^*, Javier Martínez-Sanz^1,3^, Sabina Herrera^4^, Luis Ramos-Ruperto^5^, Alejandro Díez-Vidal^5^, Talía Sainz^6^, Noelia Álvarez-Díaz^7^, Andrea Correa-Pérez^8,9,10^, Alfonso Muriel^2,10^, Jesús López-Alcalde^8,10,11^, José A. Pérez-Molina^1,2,3^, Santiago Moreno^1,2,3^ and Sergio Serrano-Villar^1,2,3^

1. Department of Infectious Diseases, Hospital Universitario Ramón y Cajal and Instituto Ramón y Cajal de Investigación Sanitaria (IRYCIS), Madrid, Spain

2. Universidad de Alcá (UAH), Madrid, Spain

3. CIBER de Enfermedades Infecciosas (CIBERINFEC), Instituto de Salud Carlos III, Madrid, Spain

4. Infectious Diseases Department, Hospital Clinic, Barcelona, Spain

5. HIV Unit, Internal Medicine Department, Hospital Universitario La Paz, Madrid, Spain

6. Pediatric Tropical and Infectious Diseases, Hospital la Paz and La Paz Research Institute (IdiPAZ), Center for Biomedical Research in Infectious Diseases (CIBERINFEC), Universidad Autónoma de Madrid, UAM, Madrid, Spain

7. Medical Library, Hospital Universitario Ramón y Cajal, Instituto Ramón y Cajal de Investigación Sanitaria (IRYCIS), Madrid, Spain

8. Faculty of Medicine, Universidad Francisco de Vitoria (UFV), Madrid, Spain

9. Pharmacy and Medical Devices Department. Hospital Central de la Defensa Gómez Ulla, Madrid, Spain

10. Clinical Biostatistics Unit, Hospital Universitario Ramón y Cajal, Instituto Ramón y Cajal de Investigación Sanitaria (IRYCIS), Centro de Investigación Biomédica en Red. Epidemiología y Salud Pública (CIBERESP), Madrid, Spain

11. Institute for Complementary and Integrative Medicine, University Hospital Zurich and University of Zurich, Zurich, Switzerland

In the published article, an author name was incorrectly written as Alejandro Díez. The correct spelling is Alejandro Díez-Vidal.

The initials used in the Author Contributions section should be AD-V.

In the published article, there was an error in the Discussion section. A correction has been made to **Discussion**, paragraph number 4. This sentence previously stated: “Also, the study by Trickey et al. (21), including patients with ≥350 CD4, found a moderately risk of all-cause mortality among subjects with high CD8+ levels (>1,138 cell/uL)”.

The corrected sentence appears below:

“Also, the study by Trickey et al. (21), including patients with ≥350 CD4, found a moderate risk of all-cause mortality among subjects with high CD8+ levels (>1,138 cell/uL)”.

In the published article, there was an error in the **Acknowledgments** section. A correction has been made to **Aknowledgments**,

At the end of the paragraph, the authors would like to add the sentence:

“This work was supported by a European Society of Pediatric Infectious Diseases (ESPID) Springboard Award”.

The authors apologize for these errors and state that this does not change the scientific conclusions of the article in any way. The original article has been updated.

