# Supplementary material for: CD4/CD8 ratio and CD8+ T-cell count as prognostic markers for non-AIDS mortality in people living with HIV. A systematic review and meta-analysis
Source: Front Immunol. 2024 Feb 1;15:1343124. doi: 10.3389/fimmu.2024.1343124 (PMC10868578; doi:10.3389/fimmu.2024.1343124)

**Supplementary material**

**Table of contents**

Page

Supplementary 1. Search strategy and studies eligibility criteria 2

Supplementary 2: QUIPS tool signaling questions 6

Supplementary 3. Data adjustment of primary studies to pre-defined cut-offs 9

Supplementary 4. Exclusion criteria 10

Supplementary 5. QUIPS template 11

Supplementary 6. Adapted Grading of Recommendations, Assessment, Development and

Evaluation (GRADE) for systematic reviews with meta-analysis of prognostic studies 19

Supplementary 7. Primary studies reporting Non-AIDS events. 20

**Supplementary 1. Search strategy and studies eligibility criteria**

| **Database** | **Web platform** | **Date** | **Results** |
| --- | --- | --- | --- |
| Medline | Ovid | 31-01-2020 | 1145 |
| Embase | Elsevier | 31-01-2020 | 2180 |
|  |  | 03-02-2020 | 275 |
| Web of Science | Fecyt |  |  |
| CENTRAL | Cochrane Database | 03-02-2020 | 180 |
| **Total** |  |  | 3780 |
| **Total without duplicated** |  |  | 2678 |

**Ovid MEDLINE(R) ALL / PubMed(R) <1946 to Present>**

1 exp HIV Infections/ (277763)

2 exp HIV/ (97412)

3 hiv.tw. (304629)

4 "hiv-1*".tw. (77892)

5 "hiv-2*".tw. (5138)

6 hiv1.tw. (871)

7 hiv2.tw. (159)

8 "hiv infect*".tw. (106102)

9 human immun#deficiency virus.tw. (84863)

10 human immun#-deficiency virus.tw. (602)

11 (human immun* adj4 deficiency virus).tw. (611)

12 acquired immun#deficiency syndrome.tw. (15802)

13 acquired immun#-deficiency syndrome.tw. (5934)

14 (acquired immun* adj4 deficiency syndrome).tw. (5976)

15 *Sexually Transmitted Diseases, Viral/ (1053)

16 1 or 2 or 3 or 4 or 5 or 6 or 7 or 8 or 9 or 10 or 11 or 12 or 13 or 14 or 15 (398335)

17 exp CD4-CD8 Ratio/ (4675)

18 exp CD8-Positive T-Lymphocytes/ (61085)

19 cd4?cd8 ratio.tw. (462)

20 cd8.tw. (87907)

21 (cd8 adj2 positive adj2 lymphocyte?).tw. (495)

22 17 or 18 or 19 or 20 or 21 (113578)

23 16 and 22 (12582)

24 exp Mortality/ (372714)

25 mortality.ti,ab. (725965)

26 dead.ti,ab. (52506)

27 death*.ti,ab. (786132)

28 died.ti,ab. (233730)

29 fatality.ti,ab. (17554)

30 fatalities.ti,ab. (10709)

31 survivor.ti,ab. (9057)

32 survival.ti,ab. (907593)

33 24 or 25 or 26 or 27 or 28 or 29 or 30 or 31 or 32 (2306685)

34 23 and 33 (1317)

35 exp Animals/ not humans.sh. (4669173)

36 34 not 35 (1191)

37 limit 36 to yr="1996 -Current" (1012)

**Embase (Elsevier)**

#38 #34 NOT #37 AND [1996-2020]/py AND [embase]/lim 1,561

#37 #35 NOT #36 5,391,331

#36 'human'/exp 21,527,103

#35 'animal'/exp 26,918,434

#34 #23 AND #33 1,992

#33 #24 OR #25 OR #26 OR #27 OR #28 OR #29 OR #30 OR #31 OR #32 3,327,701

#32 survival:ab,ti 1,336,736

#31 survivor:ab,ti 14,042

#30 fatalities:ab,ti 13,465

#29 fatality:ab,ti 22,068

#28 died:ab,ti 352,725

#27 death:ab,ti 962,880

#26 dead:ab,ti 69,753

#25 mortality:ab,ti 1,064,192

#24 'mortality'/exp 1,060,923

#23 #16 AND #22 15,811

#22 #17 OR #18 OR #19 OR #20 OR #21 149,446

#21 cd8 NEXT/2 positive NEXT/2 lymphocyte$ 688

#20 cd8:ab,ti 127,554

#19 'cd4 cd8 ratio':ab,ti 5,277

#18 'cd8+ t lymphocyte'/exp 63,468

#17 'cd4 cd8 ratio'/exp 5,267

#16 #1 OR #2 OR #3 OR #4 OR #5 OR #6 OR #7 OR #8 OR #9 OR #10 OR #11 OR #12 OR #13 OR #14 OR #15 562,259

#15 'sexually transmitted disease'/de 47,948

#14 acquired NEXT/4 immun* NEXT/4 deficiency NEXT/4 syndrome 136,545

#13 'acquired immun?-deficiency syndrome':ab,ti 6,319

#12 'acquired immun?deficiency syndrome':ab,ti 16,453

#11 human NEXT/4 immun* NEXT/4 deficiency NEXT/4 virus 1,862

#10 'human immun?-deficiency virus':ab,ti 762

#9 'human immun?deficiency virus':ab,ti 92,001

#8 'hiv infect*':ab,ti 132,847

#7 hiv2:ab,ti 246

#6 hiv1:ab,ti 1,381

#5 'hiv 2*':ab,ti 384,424

#4 'hiv 1*':ab,ti 384,424

#3 hiv:ab,ti 384,424

#2 'human immunodeficiency virus'/exp 190,547

#1 'human immunodeficiency virus infection'/exp 375,360

**Web of Science (WOS) (Search: all database except Medline)**

# 5

#4 AND #3

Bases de datos= WOS, CCC, DIIDW, KJD, RSCI, SCIELO Período de tiempo=1996-2020

Idioma de búsqueda=Auto 231

# 4

TEMA: (mortality) OR TÍTULO: (mortality OR death OR dead OR died OR fatality OR fatalities OR survivor OR survival)

Bases de datos= WOS, CCC, DIIDW, KJD, RSCI, SCIELO Período de tiempo=1996-2020

Idioma de búsqueda=Auto 1.746.935

# 3

#2 AND #1

Bases de datos= WOS, CCC, DIIDW, KJD, RSCI, SCIELO Período de tiempo=1996-2020

Idioma de búsqueda=Auto 3.627

# 2

TEMA: ("cd4-cd8 ratio" OR "cd8 positive t lymphocites") OR TÍTULO: ("cd4-cd8 ratio" OR cd8 positive t lymphocites OR cd8)

Bases de datos= WOS, CCC, DIIDW, KJD, RSCI, SCIELO Período de tiempo=1996-2020

Idioma de búsqueda=Auto 30.204

# 1

TEMA: ("hiv infections" OR "human immunodeficiency virus" OR "acquired immuno deficiency syndrome") OR TÍTULO: (hiv infec* OR hiv)

Bases de datos= WOS, CCC, DIIDW, KJD, RSCI, SCIELO Período de tiempo=1996-2020

Idioma de búsqueda=Auto 331.889

**CENTRAL (Cochrane Library)**

#1 MeSH descriptor: [HIV Infections] explode all trees 12234

#2 MeSH descriptor: [HIV] explode all trees 3013

#3 (hiv*):ti,ab,kw (Word variations have been searched) 26146

#4 (hiv-1):ti,ab,kw 6324

#5 (hiv-2):ti,ab,kw 103

#6 (hiv NEXT infect*):ti,ab,kw 15677

#7 ("human immunodeficiency virus"):ti,ab,kw 11760

#8 ("acquired immunodeficiency syndrome"):ti,ab,kw 2224

#9 MeSH descriptor: [Sexually Transmitted Diseases, Viral] this term only 27

#10 #1 or #2 or #3 or #4 or #5 or #6 or #7 or #8 or #9 27674

#11 MeSH descriptor: [CD4-CD8 Ratio] explode all trees 318

#12 MeSH descriptor: [CD8-Positive T-Lymphocytes] explode all trees 663

#13 (cd4?cd8 ratio):ti,ab,kw 799

#14 (cd8):ti,ab,kw 4608

#15 (cd8 NEXT positive NEXT lymphocyte?):ti,ab,kw 8

#16 #11 or #12 or #13 or #14 or #15 4704

#17 #10 and #16 1101

#18 MeSH descriptor: [Mortality] explode all trees 13049

#19 (mortality):ti,ab,kw 91777

#20 (dead):ti,ab,kw 1721

#21 (death*):ti,ab,kw 70306

#22 (died):ti,ab,kw 13520

#23 (fatality):ti,ab,kw 3235

#24 (fatalities):ti,ab,kw 244

#25 (survivor):ti,ab,kw 3995

#26 (survival):ti,ab,kw 104670

#27 #19 or #20 or #21 or #22 or #23 or #24 or #25 or #26 207249

#28 #17 and #27 149

#29 MeSH descriptor: [Animals] explode all trees 15783

#30 MeSH descriptor: [Humans] explode all trees 8477

#31 #29 NOT #30 7306

#32 #28 NOt #31 in Trials 148

**INCLUSION CRITERIA AND STUDIES ELIGIBILITY**

- Experimental/observational studies

- Patients over 18 years of age

- HIV 1 or 2 on ART with undetectable VL

- CD4/CD8 ratio or CD8 count and non-AIDS mortality endpoint

- CD4/CD8 ratio or CD8 count and all-cause mortality endpoint

- CD4/CD8 ratio or CD8 count and non-AIDS events endpoint

- ART trials with mortality or non-AIDS events endpoints

- Studies with oncologic patients and chemotherapy/immunosuppressive treatments

**EXCLUSION CRITERIA**

- Case reports/series

- Studies with elite controllers

### Supplementary 2: QUIPS tool signaling questions

| **Signalling question** | **Authors' judgement for 'yes'** |
| --- | --- |
| **Study participation** |  |
| a. Adequate participation in the study by eligible persons | The study sample was consecutively recruited from the target population, or, for case‐control studies, the cases and controls were from similar populations |
| b. Description of the target population or population of interest | Source population for adults with HIV on ART and undetectable VL |
| c. Description of the baseline study sample | Number of participants on ART and undetectable viral load, measurement of baseline ratio (table 1) |
| d. Adequate description of the sampling frame and recruitment | Establishment of target population, selection criteria and key characteristics of the target population clearly described |
| e. Adequate description of the period and place of recruitment | Period and place of recruitment for both baseline and follow‐up are clearly described.  Years of follow up. Time relationship between ratio measurement and outcome |
| f. Adequate description of inclusion and exclusion criteria | Inclusion of PWHIV on ART and undetectable VL, exclusion of AIDS or people without treatment |
| **Study participation: risk of bias rating (high/low/unclear)** | **High**: most items are answered with 'no'; **Low**: most items answered with 'yes'; **Moderate**: most items are answered with 'moderate'  Note: potentially a single item may introduce a high risk of bias, depending on study specifics |
| **Study attrition: yes/no/unclear/NA** |  |
| a. Adequate response rate for study participants | Cut-off 10% |
| b. Description of attempts to collect information on participants who dropped out | Attempts to collect information on participants who dropped out are provided |
| c. Reasons for loss to follow‐up provided | Reasons on participants who dropped out are provided |
| d. Adequate description of participants lost to follow‐up | Description provided |
| e. No important differences between participants who completed the study and those who did not | Study authors described differences between participants who completed the study and those who did not as not important or information provided to judge the differences. |
| **Study attrition: risk of bias rating (high/low/unclear)** | **High:** most items are answered with 'no'; **Low:** most items answered with 'yes'**; Moderate:** most items are answered with 'moderate'  Note: potentially a single item may introduce a high risk of bias, depending on study specifics |
| **Prognostic factor measurement: yes/no/unclear/NA** |  |
| a. Clear definition or description provided | CD4/CD8 ratio or CD8 measurement |
| b. Adequately valid and reliable method of measurement | Laboratory method, data recruitment |
| c. Continuous variables reported or appropriate cut points used | Report of cutoff points, fitted variable described, |
| d. Same method and setting of measurement used in all study participants |  |
|  |  |
| f. Appropriate methods of imputation were used for missing data | Reported imputation of missing data |
| **Ratio measurement: risk of bias rating (high/low/unclear)** |  |
| **Outcome measurement: yes/no/unclear**  **Outcome measurement:** **yes/no/unclear/NA** |  |
| a. Clear definition of the outcome provided | Measurement of mortality has to be defined (including duration of follow‐up) |
| b. Adequately valid and reliable method of outcome measurement | Standardized databases |
| c. Same method and setting of outcome measurement used in all study participants | Method described |
| **Outcome measurement: risk of bias rating (high/low/unclear)** | **High:** items *a* is answered with 'no'; **Low:** items *a* is answered with 'yes'**; Moderate**: most items are answered with 'moderate' |
| **Adjustment for other prognostic factors: yes/no/unclear** |  |
| a. Measurement of all other important PFs measured. Covariables | Core set of adjustment factors: Age, sex, CD4 nadir, risk factor for HIV transmission, HCV serostatus, CMV serostatus, type of ART |
| b. Provision of clear definitions of important PFs measured | Measurement of core set of adjustment factors has to be clearly described |
| c. Adequately valid and reliable measurement of all important PFs | Measurement of core set of adjustment factors is valid and reliable |
| d. Use of same method and setting of PFs measurement in all study participants | Measurements of core set of adjustment factors are the same for all study participants |
| e. Appropriate imputation methods used for missing PFs (if applicable) | Strategy to impute missing core set of adjustment factors data is described |
| f. Important PFs were accounted for in the study design. | Methods section of the publication describes strategy to account for core set of adjustment factors |
| g. Important PFs were accounted for in the analysis. | Important core set of adjustment factors are accounted for in multivariable logistic regression and Cox proportional hazards models, or adjusted and unadjusted analyses were compared, and differences identified |
| **Adjustment for other prognostic factors: risk of bias rating (high/low/unclear)** | **High:** most items are answered with 'no'; **Low:** most items answered with 'yes'**; Moderate**: most items are answered with 'moderate'  Note: potentially a single item may introduce a high risk of bias, depending on study specifics |
| **Statistical analysis and reporting: yes/no/unclear/NA** |  |
| a. Sufficient presentation of data to assess the adequacy of the analytic strategy. | Measures of association to analyze in selected studies will include OR, RR and HR, including its standard error or confidence intervals (CI). |
| b. Strategy for model building is appropriate and based on a conceptual framework or model. | NA: we do not anticipate conceptual frameworks or model building strategies for this type of research question (focusing on one prognostic factor only) |
| c. Statistical model is adequate for the study design. | Mainly incidence rates, uni‐ and multivariate logistic regression, Cox proportional hazard model |
| d. No selective reporting of results | Publication of protocol or registration submitted through public database |
| **Statistical analysis and reporting: risk of bias rating (high/low/unclear)** | **High:** most items are answered with 'no'; **Low:** most items answered with 'yes'**; Moderate**: most items are answered with 'moderate'  Note: potentially a single item may introduce a high risk of bias, depending on study specifics |

**No**: No or no relevant information to answer the signaling question. **Moderate**: insufficient information to make a judgment of signaling question of yes or no
**NA** (not applicable): signaling question not appropriate for this type of prognostic review

NRT: non-randomized controlled study

RTC: randomized clinical trials

PF: prognostic factor

**Supplementary 3. Data adjustment of primary studies to pre-defined cut-offs**

In the study conducted by Novak et al. (41), we sought data that would allow us to estimate the effect of the ratio, in this instance using 0.5 as the cut-off point. For the research by Trickey et al. (21), we requested an adjusted data analysis with a CD4/CD8 ratio cut-off point of 0.4. In the case of the study by Boettiger et al. (39), we computed the mortality odds ratio (OR) for a ratio cut-off of less than 0.4. For the studies authored by Han et al., and Serrano-Villar et al. (19, 20), which presented ratio data in tertiles, we executed an initial meta-analysis for each study to calculate the HR or OR for the lower vs. higher CD4/CD8 ratio categories at the 0.30-0.45 cut-offs. The computed effects were then included in the final meta-analysis (see Figures 1 and 2 below).

**Figure 1. Meta-analysis of low CD4/CD8 ratio categories versus high for the study by Han et al.**


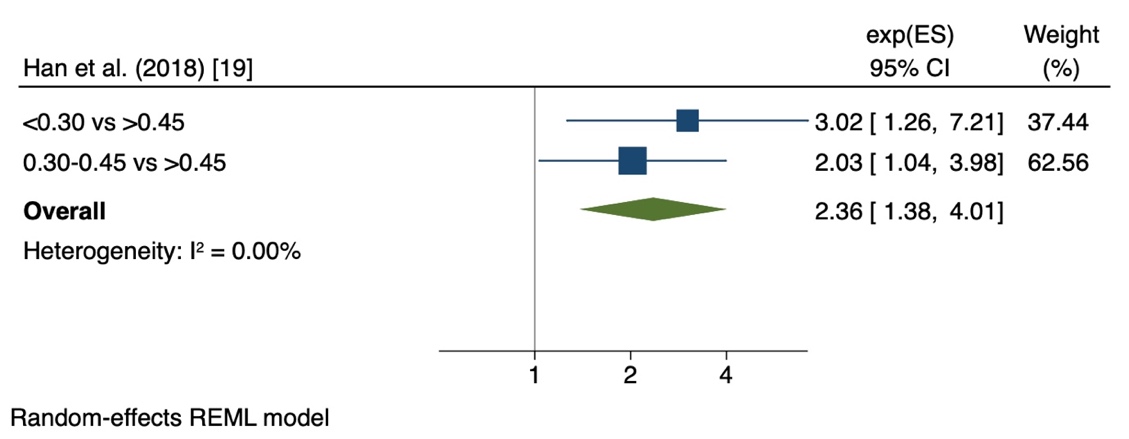


**Figure 1. Meta-analysis of low CD4/CD8 ratio categories versus high for the study by Serrano-Villar et al.**


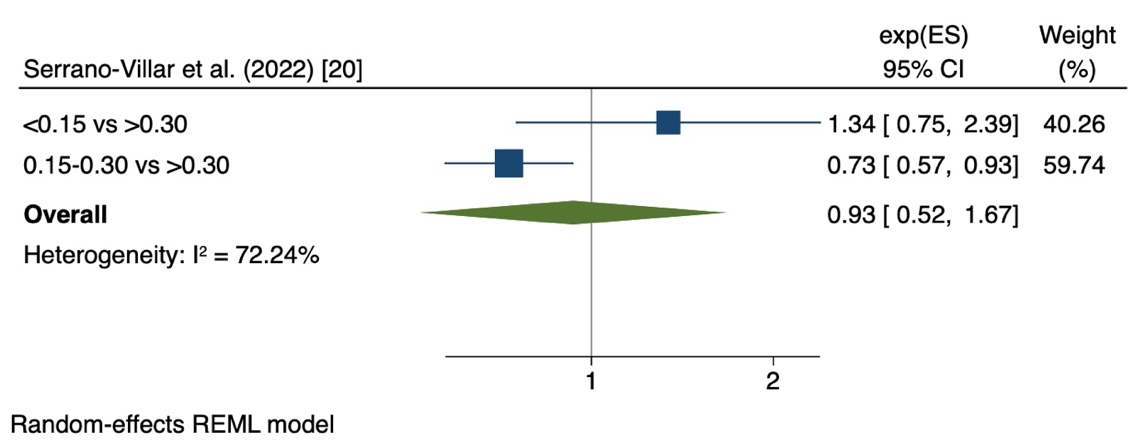


**Supplementary 4. Exclusion criteria**

**Studies included in the systematic review but excluded from the meta-analysis**

Three studies included part of the population with detectable viral load (28,29,30). The study by Liu et al. (31) assessed the impact on mortality of CD8 and CD4/CD8 ratio values determined at baseline before treatment, and thus before adequate virological control. Similarly, the studies by Domínguez et al. (33) and Helleberg et al. (32) started monitoring patients before treatment initiation with detectable baseline viral loads. We excluded the study by Domínguez et al. (33) from the meta-analysis because it was based on the same cohort as the study by Martínez-Sanz et al. (35), contributing to the same outcomes. We selected the study by Martínez-Sanz et al. as it was focused on non-AIDS events, was more recent, and with a longer follow-up period. We also excluded the study by Lee et al. (37) from the meta-analysis because the CD4/CD8 ratio was measured at 6 months from ART initiation, corresponding to an early recovery stage of the biomarker. Three studies reported the ratio measurement as a continuous variable, but we were unable to conduct a meta-analysis of these values due to methodological differences between them and high heterogeneity in the assessment of the coefficient to evaluate (9,29,43). We finally selected four studies for the meta-analysis including “non-AIDS” or “all-cause mortality” outcomes (18,35,39,41), and seven studies for “non-AIDS mortality”, “all-cause mortality”, and composite outcomes (18-21,35,39,41). This scarcity was due to the high variability across studies, mainly the differences in the cut-off points of the CD4/CD8 ratio and CD8+ counts.

**Systematic-review exclusion**

Among the reasons for exclusion were studies focused on AIDS mortality, studies with a higher proportion of patients with detectable viral load or incomplete data on ART or virological suppression, and studies assessing lymphocyte subpopulations, but not reporting CD4/CD8 ratio or CD8+ total counts. We found two conference communications that met eligibility criteria (see references below 1,2), but they were not included in the systematic review due to incomplete data, differences in CD4/CD8 ratio categorization, and CD8 T-cell reference values.

**References. Congress communications.**

1. Keith Sigel, Kristina Crothers, Sheldon T. Brown, et al., Treatment and Outcomes of Non-Small Cell Lung Cancer in Later Antiretroviral-Era HIV Infection. Presented at Conference on Retroviruses and Opportunistic Infections (CROI) 2017.

2. Stefan Esser, Norbert H. Brockmeyer, Martin Hower, et al., Association between Cardiovascular Events and HIV-specific Risk Factors. Presented at Conference on Retroviruses and Opportunistic Infections (CROI) 2017.

**Supplementary 5. QUIPS template**

|  |  |  |  |  |  |  | **Serrano-Villar 2014*** | **Serrano-Villar 2014**** | **Helleberg 2015** |
| --- | --- | --- | --- | --- | --- | --- | --- | --- | --- |
| **Study participation** | |  |  |  |  |  |  |  |  |
| a. Adequate participation in the study by eligible persons | | | | |  |  | Yes | Yes | Yes |
| b. Description of the target population or population of interest | | | | |  |  | Yes | Yes | Yes |
| c. Description of the baseline study sample | | | |  |  |  | Yes | Yes | Yes |
| d. Adequate description of the sampling frame and recruitment | | | | |  |  | Yes | Yes | Yes |
| e. Adequate description of the period and place of recruitment | | | | |  |  | Yes | No | Yes |
| f. Adequate description of inclusion and exclusion criteria | | | | |  |  | Yes | No | Yes |
| **Risk of bias** |  |  |  |  |  |  | **Low** | **Low** | **Low** |
| **Study attrition** | |  |  |  |  |  |  |  |  |
| a. Adequate response rate for study participants | | | |  |  |  | No | Moderate | Moderate |
| b. Description of attempts to collect information on participants who dropped out | | | | | |  | No | No | No |
| c. Reasons for loss to follow‐up provided | | |  |  |  |  | No | No | No |
| d. Adequate description of participants lost to follow‐up | | | |  |  |  | No | No | No |
| e. No important differences between participants who completed the study and those who did not | | | | | | | No | Moderate | Moderate |
| **Risk of bias** |  |  |  |  |  |  | **High** | **High** | **High** |
| **Prognostic factor measurement** | | |  |  |  |  |  |  |  |
| a. Clear definition or description provided | | |  |  |  |  | Yes | Yes | Yes |
| b. Adequately valid and reliable method of measurement | | | | |  |  | Yes | Yes | Yes |
| c. Continuous variables reported or appropriate cut points used | | | | |  |  | Yes | Yes | Yes |
| d. Same method and setting of measurement used in all study participants | | | | | |  | Yes | Yes | Yes |
| e. Appropriate methods of imputation were used for missing data | | | | |  |  | No | Yes | No |
| **Risk of bias** |  |  |  |  |  |  | **Low** | **Low** | **Low** |
| **Outcome measurement** | |  |  |  |  |  |  |  |  |
| a. Clear definition of the outcome provided | | | |  |  |  | Moderate | Moderate | Moderate |
| b. Adequately valid and reliable method of outcome measurement | | | | |  |  | Yes | Yes | Yes |
| c. Same method and setting of outcome measurement used in all study participants | | | | | |  | Yes | Yes | Yes |
| **Risk of bias** |  |  |  |  |  |  | **Low** | **Low** | **Low** |
| **Adjustment for other prognostic factors** | | |  |  |  |  |  |  |  |
| a. Measurement of all other important PFs measured | | | |  |  |  | No | No | No |
| b. Provision of clear definitions of important PFs measured | | | | |  |  | Yes | Yes | Yes |
| c. Adequately valid and reliable measurement of all important PFs | | | | |  |  | Yes | Yes | Yes |
| d. Use of same method and setting of PFs measurement in all study participants | | | | | |  | Yes | Yes | Yes |
| e. Appropriate imputation methods used for missing PFs | | | | |  |  | Yes | Moderate | No |
| f. Important PFs were accounted for in the study design | | | |  |  |  | Yes | Yes | Yes |
| g. Important PFs were accounted for in the analysis | | | |  |  |  | Yes | Yes | Yes |
| **Risk of bias** |  |  |  |  |  |  | **Low** | **Low** | **Low** |
| **Statistical analysis and reporting** | | |  |  |  |  |  |  |  |
| a. Sufficient presentation of data to assess the adequacy of the analytic strategy | | | | | |  | Yes | Yes | No |
| b. Strategy for model building is appropriate and based on a conceptual framework or model | | | | | | | Yes | Yes | Yes |
| c. Statistical model is adequate for the study design | | | |  |  |  | Yes | Yes | Moderate |
| d. No selective reporting of results | | |  |  |  |  | Moderate | Moderate | Moderate |
| **Risk of bias** |  |  |  |  |  |  | **Moderate** | **Moderate** | **Moderate** |
| **Overall risk of bias** | |  |  |  |  |  | **MODERATE** | **MODERATE** | **MODERATE** |
| * Serrano-Villar Plos One 2014 (18). ** Serrano-Villar Plos Pathogens 2014 (9) | | | | | |  |  |  |  |

**Supplementary 5. QUIPS template cont.**

|  |  |  |  |  |  |  | **Mussini 2015** | **Cervero 2016** | **Collin 2016** |
| --- | --- | --- | --- | --- | --- | --- | --- | --- | --- |
| **Study participation** | |  |  |  |  |  |  |  |  |
| a. Adequate participation in the study by eligible persons | | | | |  |  | Yes | No | Yes |
| b. Description of the target population or population of interest | | | | |  |  | Yes | Yes | Yes |
| c. Description of the baseline study sample | | | |  |  |  | Yes | Yes | Yes |
| d. Adequate description of the sampling frame and recruitment | | | | |  |  | Yes | No | Yes |
| e. Adequate description of the period and place of recruitment | | | | |  |  | Yes | Yes | Yes |
| f. Adequate description of inclusion and exclusion criteria | | | | |  |  | Yes | No | No |
| **Risk of bias** |  |  |  |  |  |  | **Low** | **Moderate** | **Low** |
| **Study attrition** | |  |  |  |  |  |  |  |  |
| a. Adequate response rate for study participants | | | |  |  |  | Yes | Moderate | Moderate |
| b. Description of attempts to collect information on participants who dropped out | | | | | |  | Yes | No | Yes |
| c. Reasons for loss to follow‐up provided | | |  |  |  |  | Yes | No | No |
| d. Adequate description of participants lost to follow‐up | | | |  |  |  | Yes | No | No |
| e. No important differences between participants who completed the study and those who did not | | | | | | | Moderate | Moderate | No |
| **Risk of bias** |  |  |  |  |  |  | **Low** | **High** | **High** |
| **Prognostic factor measurement** | | |  |  |  |  |  |  |  |
| a. Clear definition or description provided | | |  |  |  |  | Yes | Moderate | Moderate |
| b. Adequately valid and reliable method of measurement | | | | |  |  | Yes | Yes | Moderate |
| c. Continuous variables reported or appropriate cut points used | | | | |  |  | Yes | Yes | Yes |
| d. Same method and setting of measurement used in all study participants | | | | | |  | Yes | Yes | Moderate |
| e. Appropriate methods of imputation were used for missing data | | | | |  |  | No | No | No |
| **Risk of bias** |  |  |  |  |  |  | **Low** | **Low** | **Moderate** |
| **Outcome measurement** | |  |  |  |  |  |  |  |  |
| a. Clear definition of the outcome provided | | | |  |  |  | Moderate | Moderate | Moderate |
| b. Adequately valid and reliable method of outcome measurement | | | | |  |  | Yes | Yes | Yes |
| c. Same method and setting of outcome measurement used in all study participants | | | | | |  | Yes | Yes | Yes |
| **Risk of bias** |  |  |  |  |  |  | **Low** | **Low** | **Low** |
| **Adjustment for other prognostic factors** | | |  |  |  |  |  |  |  |
| a. Measurement of all other important PFs measured | | | |  |  |  | Yes | No | No |
| b. Provision of clear definitions of important PFs measured | | | | |  |  | Yes | No | Yes |
| c. Adequately valid and reliable measurement of all important PFs | | | | |  |  | Yes | No | Moderate |
| d. Use of same method and setting of PFs measurement in all study participants | | | | | |  | Yes | Yes | Yes |
| e. Appropriate imputation methods used for missing PFs | | | | |  |  | No | No | No |
| f. Important PFs were accounted for in the study design | | | |  |  |  | Yes | No | Yes |
| g. Important PFs were accounted for in the analysis | | | |  |  |  | Yes | No | Yes |
| **Risk of bias** |  |  |  |  |  |  | **Low** | **High** | **Low** |
| **Statistical analysis and reporting** | | |  |  |  |  |  |  |  |
| a. Sufficient presentation of data to assess the adequacy of the analytic strategy | | | | | |  | Yes | No | No |
| b. Strategy for model building is appropriate and based on a conceptual framework or model | | | | | | | Yes | No | No |
| c. Statistical model is adequate for the study design | | | |  |  |  | Yes | No | No |
| d. No selective reporting of results | | |  |  |  |  | Yes | Moderate | Moderate |
| **Risk of bias** |  |  |  |  |  |  | **Low** | **High** | **High** |
| **Overall risk of bias** | |  |  |  |  |  | **LOW** | **HIGH** | **HIGH** |

**Supplementary 5. QUIPS template cont.**

|  |  |  |  |  |  |  | **Lee 2017** | **Trickey 2017** | **Duffau 2018** |
| --- | --- | --- | --- | --- | --- | --- | --- | --- | --- |
| **Study participation** | |  |  |  |  |  |  |  |  |
| a. Adequate participation in the study by eligible persons | | | | |  |  | Moderate | Yes | Yes |
| b. Description of the target population or population of interest | | | | |  |  | Yes | Yes | Yes |
| c. Description of the baseline study sample | | | |  |  |  | Yes | Yes | Yes |
| d. Adequate description of the sampling frame and recruitment | | | | |  |  | Yes | Yes | Yes |
| e. Adequate description of the period and place of recruitment | | | | |  |  | Yes | Yes | Yes |
| f. Adequate description of inclusion and exclusion criteria | | | | |  |  | Yes | No | Yes |
| **Risk of bias** |  |  |  |  |  |  | **Moderate** | **Low** | **Low** |
| **Study attrition** | |  |  |  |  |  |  |  |  |
| a. Adequate response rate for study participants | | | |  |  |  | Yes | Yes | Moderate |
| b. Description of attempts to collect information on participants who dropped out | | | | | |  | No | No | No |
| c. Reasons for loss to follow‐up provided | | |  |  |  |  | No | No | No |
| d. Adequate description of participants lost to follow‐up | | | |  |  |  | No | No | No |
| e. No important differences between participants who completed the study and those who did not | | | | | | | Moderate | Moderate | Moderate |
| **Risk of bias** |  |  |  |  |  |  | **High** | **High** | **High** |
| **Prognostic factor measurement** | | |  |  |  |  |  |  |  |
| a. Clear definition or description provided | | |  |  |  |  | Yes | Yes | Moderate |
| b. Adequately valid and reliable method of measurement | | | | |  |  | Yes | Yes | Yes |
| c. Continuous variables reported or appropriate cut points used | | | | |  |  | Yes | Yes | Yes |
| d. Same method and setting of measurement used in all study participants | | | | | |  | Yes | Yes | Yes |
| e. Appropriate methods of imputation were used for missing data | | | | |  |  | No | Moderate | Moderate |
| **Risk of bias** |  |  |  |  |  |  | **Low** | **Low** | **Low** |
| **Outcome measurement** | |  |  |  |  |  |  |  |  |
| a. Clear definition of the outcome provided | | | |  |  |  | Moderate | Moderate | Yes |
| b. Adequately valid and reliable method of outcome measurement | | | | |  |  | Yes | Yes | Moderate |
| c. Same method and setting of outcome measurement used in all study participants | | | | | |  | No | Yes | Yes |
| **Risk of bias** |  |  |  |  |  |  | **Low** | **Low** | **Low** |
| **Adjustment for other prognostic factors** | | |  |  |  |  |  |  |  |
| a. Measurement of all other important PFs measured | | | |  |  |  | No | No | No |
| b. Provision of clear definitions of important PFs measured | | | | |  |  | Yes | Yes | Yes |
| c. Adequately valid and reliable measurement of all important PFs | | | | |  |  | Moderate | No | Yes |
| d. Use of same method and setting of PFs measurement in all study participants | | | | | |  | Moderate | Moderate | Yes |
| e. Appropriate imputation methods used for missing PFs | | | | |  |  | Moderate | Moderate | Moderate |
| f. Important PFs were accounted for in the study design | | | |  |  |  | No | Yes | Yes |
| g. Important PFs were accounted for in the analysis | | | |  |  |  | No | Yes | Yes |
| **Risk of bias** |  |  |  |  |  |  | **Moderate** | **Low** | **Low** |
| **Statistical analysis and reporting** | | |  |  |  |  |  |  |  |
| a. Sufficient presentation of data to assess the adequacy of the analytic strategy | | | | | |  | Yes | Yes | Yes |
| b. Strategy for model building is appropriate and based on a conceptual framework or model | | | | | | | Yes | Yes | Yes |
| c. Statistical model is adequate for the study design | | | |  |  |  | Yes | Yes | Yes |
| d. No selective reporting of results | | |  |  |  |  | Moderate | Yes | Moderate |
| **Risk of bias** |  |  |  |  |  |  | **Moderate** | **Low** | **Moderate** |
| **Overall risk of bias** | |  |  |  |  |  | **HIGH** | **MODERATE** | **MODERATE** |

**Supplementary 5. QUIPS template cont.**

|  |  |  |  |  |  |  | **Han 2018** | **Castilho 2019** | **Aldrete 2020** |
| --- | --- | --- | --- | --- | --- | --- | --- | --- | --- |
| **Study participation** | |  |  |  |  |  |  |  |  |
| a. Adequate participation in the study by eligible persons | | | | |  |  | Yes | Yes | Yes |
| b. Description of the target population or population of interest | | | | |  |  | Yes | Yes | Yes |
| c. Description of the baseline study sample | | | |  |  |  | Yes | Yes | Yes |
| d. Adequate description of the sampling frame and recruitment | | | | |  |  | Yes | Yes | Yes |
| e. Adequate description of the period and place of recruitment | | | | |  |  | Yes | Yes | No |
| f. Adequate description of inclusion and exclusion criteria | | | | |  |  | Yes | Yes | No |
| **Risk of bias** |  |  |  |  |  |  | **Low** | **Low** | **Low** |
| **Study attrition** | |  |  |  |  |  |  |  |  |
| a. Adequate response rate for study participants | | | |  |  |  | Moderate | Moderate | Moderate |
| b. Description of attempts to collect information on participants who dropped out | | | | | |  | No | No | No |
| c. Reasons for loss to follow‐up provided | | |  |  |  |  | No | No | No |
| d. Adequate description of participants lost to follow‐up | | | |  |  |  | No | No | No |
| e. No important differences between participants who completed the study and those who did not | | | | | | | Moderate | Moderate | No |
| **Risk of bias** |  |  |  |  |  |  | **High** | **High** | **High** |
| **Prognostic factor measurement** | | |  |  |  |  |  |  |  |
| a. Clear definition or description provided | | |  |  |  |  | Moderate | Yes | Yes |
| b. Adequately valid and reliable method of measurement | | | | |  |  | Yes | Yes | Yes |
| c. Continuous variables reported or appropriate cut points used | | | | |  |  | Yes | Yes | Yes |
| d. Same method and setting of measurement used in all study participants | | | | | |  | Yes | Yes | Yes |
| e. Appropriate methods of imputation were used for missing data | | | | |  |  | Moderate | Moderate | No |
| **Risk of bias** |  |  |  |  |  |  | **Low** | **Low** | **Low** |
| **Outcome measurement** | |  |  |  |  |  |  |  |  |
| a. Clear definition of the outcome provided | | | |  |  |  | Moderate | Moderate | Moderate |
| b. Adequately valid and reliable method of outcome measurement | | | | |  |  | Yes | Yes | Yes |
| c. Same method and setting of outcome measurement used in all study participants | | | | | |  | Yes | Yes | Yes |
| **Risk of bias** |  |  |  |  |  |  | **Low** | **Low** | **Low** |
| **Adjustment for other prognostic factors** | | |  |  |  |  |  |  |  |
| a. Measurement of all other important PFs measured | | | |  |  |  | No | No | No |
| b. Provision of clear definitions of important PFs measured | | | | |  |  | Yes | Yes | No |
| c. Adequately valid and reliable measurement of all important PFs | | | | |  |  | Moderate | Yes | Moderate |
| d. Use of same method and setting of PFs measurement in all study participants | | | | | |  | Yes | Yes | Moderate |
| e. Appropriate imputation methods used for missing PFs | | | | |  |  | Moderate | Yes | No |
| f. Important PFs were accounted for in the study design | | | |  |  |  | Yes | Yes | No |
| g. Important PFs were accounted for in the analysis | | | |  |  |  | Yes | Yes | Yes |
| **Risk of bias** |  |  |  |  |  |  | **Moderate** | **Low** | **High** |
| **Statistical analysis and reporting** | | |  |  |  |  |  |  |  |
| a. Sufficient presentation of data to assess the adequacy of the analytic strategy | | | | | |  | Yes | Yes | Yes |
| b. Strategy for model building is appropriate and based on a conceptual framework or model | | | | | | | Yes | Yes | Yes |
| c. Statistical model is adequate for the study design | | | |  |  |  | Yes | Yes | Yes |
| d. No selective reporting of results | | |  |  |  |  | Moderate | Moderate | Moderate |
| **Risk of bias** |  |  |  |  |  |  | **Moderate** | **Moderate** | **Moderate** |
| **Overall risk of bias** | |  |  |  |  |  | **MODERATE** | **MODERATE** | **HIGH** |
|  |  |  |  |  |  |  |  |  |  |

**Supplementary 5. QUIPS template cont.**

|  |  |  |  |  |  |  | **Boettiger 2020** | **Liu 2020** | **Klugman 2021** |
| --- | --- | --- | --- | --- | --- | --- | --- | --- | --- |
| **Study participation** | |  |  |  |  |  |  |  |  |
| a. Adequate participation in the study by eligible persons | | | | |  |  | Yes | Moderate | Moderate |
| b. Description of the target population or population of interest | | | | |  |  | Yes | Yes | Yes |
| c. Description of the baseline study sample | | | |  |  |  | Yes | Yes | Yes |
| d. Adequate description of the sampling frame and recruitment | | | | |  |  | Yes | Yes | Yes |
| e. Adequate description of the period and place of recruitment | | | | |  |  | Yes | Yes | Yes |
| f. Adequate description of inclusion and exclusion criteria | | | | |  |  | No | Yes | Yes |
| **Risk of bias** |  |  |  |  |  |  | **Low** | **Moderate** | **Moderate** |
| **Study attrition** | |  |  |  |  |  |  |  |  |
| a. Adequate response rate for study participants | | | |  |  |  | Moderate | Moderate | Moderate |
| b. Description of attempts to collect information on participants who dropped out | | | | | |  | No | No | No |
| c. Reasons for loss to follow‐up provided | | |  |  |  |  | No | No | No |
| d. Adequate description of participants lost to follow‐up | | | |  |  |  | No | No | No |
| e. No important differences between participants who completed the study and those who did not | | | | | | | No | Moderate | Moderate |
| **Risk of bias** |  |  |  |  |  |  | **High** | **High** | **High** |
| **Prognostic factor measurement** | | |  |  |  |  |  |  |  |
| a. Clear definition or description provided | | |  |  |  |  | Moderate | Yes | Yes |
| b. Adequately valid and reliable method of measurement | | | | |  |  | Yes | Yes | Yes |
| c. Continuous variables reported or appropriate cut points used | | | | |  |  | Yes | Yes | Yes |
| d. Same method and setting of measurement used in all study participants | | | | | |  | Yes | Moderate | Yes |
| e. Appropriate methods of imputation were used for missing data | | | | |  |  | Moderate | Moderate | Yes |
| **Risk of bias** |  |  |  |  |  |  | **Low** | **Low** | **Low** |
| **Outcome measurement** | |  |  |  |  |  |  |  |  |
| a. Clear definition of the outcome provided | | | |  |  |  | Moderate | Moderate | Moderate |
| b. Adequately valid and reliable method of outcome measurement | | | | |  |  | Yes | Moderate | Yes |
| c. Same method and setting of outcome measurement used in all study participants | | | | | |  | Yes | Moderate | No |
| **Risk of bias** |  |  |  |  |  |  | **Low** | **Moderate** | **Moderate** |
| **Adjustment for other prognostic factors** | | |  |  |  |  |  |  |  |
| a. Measurement of all other important PFs measured | | | |  |  |  | No | No | No |
| b. Provision of clear definitions of important PFs measured | | | | |  |  | Yes | No | Yes |
| c. Adequately valid and reliable measurement of all important PFs | | | | |  |  | Moderate | Moderate | Moderate |
| d. Use of same method and setting of PFs measurement in all study participants | | | | | |  | Yes | Yes | Yes |
| e. Appropriate imputation methods used for missing PFs | | | | |  |  | No | Moderate | Moderate |
| f. Important PFs were accounted for in the study design | | | |  |  |  | Yes | Moderate | Yes |
| g. Important PFs were accounted for in the analysis | | | |  |  |  | Yes | Yes | Yes |
| **Risk of bias** |  |  |  |  |  |  | **Low** | **Moderate** | **Low** |
| **Statistical analysis and reporting** | | |  |  |  |  |  |  |  |
| a. Sufficient presentation of data to assess the adequacy of the analytic strategy | | | | | |  | Yes | Yes | Yes |
| b. Strategy for model building is appropriate and based on a conceptual framework or model | | | | | | | Yes | Yes | Yes |
| c. Statistical model is adequate for the study design | | | |  |  |  | Yes | Yes | Yes |
| d. No selective reporting of results | | |  |  |  |  | Moderate | Moderate | Moderate |
| **Risk of bias** |  |  |  |  |  |  | **Moderate** | **Moderate** | **Moderate** |
| **Overall risk of bias** | |  |  |  |  |  | **MODERATE** | **HIGH** | **HIGH** |

**Supplementary 5. QUIPS template cont.**

|  |  |  |  |  |  |  | **Aksak-Wąs 2022** | **Domínguez 2022** | **Novak 2022** |
| --- | --- | --- | --- | --- | --- | --- | --- | --- | --- |
| **Study participation** | |  |  |  |  |  |  |  |  |
| a. Adequate participation in the study by eligible persons | | | | |  |  | Yes | Moderate | Yes |
| b. Description of the target population or population of interest | | | | |  |  | Yes | Yes | Yes |
| c. Description of the baseline study sample | | | |  |  |  | Yes | Yes | Yes |
| d. Adequate description of the sampling frame and recruitment | | | | |  |  | Yes | Yes | Yes |
| e. Adequate description of the period and place of recruitment | | | | |  |  | Yes | Yes | Yes |
| f. Adequate description of inclusion and exclusion criteria | | | | |  |  | Yes | Yes | Yes |
| **Risk of bias** |  |  |  |  |  |  | **Low** | **Moderate** | **Low** |
| **Study attrition** | |  |  |  |  |  |  |  |  |
| a. Adequate response rate for study participants | | | |  |  |  | Moderate | Moderate | Yes |
| b. Description of attempts to collect information on participants who dropped out | | | | | |  | No | No | No |
| c. Reasons for loss to follow‐up provided | | |  |  |  |  | No | No | No |
| d. Adequate description of participants lost to follow‐up | | | |  |  |  | No | No | No |
| e. No important differences between participants who completed the study and those who did not | | | | | | | Moderate | Moderate | Moderate |
| **Risk of bias** |  |  |  |  |  |  | **High** | **High** | **High** |
| **Prognostic factor measurement** | | |  |  |  |  |  |  |  |
| a. Clear definition or description provided | | |  |  |  |  | Yes | Moderate | Moderate |
| b. Adequately valid and reliable method of measurement | | | | |  |  | Yes | Yes | Yes |
| c. Continuous variables reported or appropriate cut points used | | | | |  |  | Yes | Yes | Yes |
| d. Same method and setting of measurement used in all study participants | | | | | |  | Yes | Yes | Yes |
| e. Appropriate methods of imputation were used for missing data | | | | |  |  | Moderate | Moderate | Moderate |
| **Risk of bias** |  |  |  |  |  |  | **Low** | **Low** | **Low** |
| **Outcome measurement** | |  |  |  |  |  |  |  |  |
| a. Clear definition of the outcome provided | | | |  |  |  | Yes | Moderate | Moderate |
| b. Adequately valid and reliable method of outcome measurement | | | | |  |  | Yes | Yes | Yes |
| c. Same method and setting of outcome measurement used in all study participants | | | | | |  | Yes | Yes | Yes |
| **Risk of bias** |  |  |  |  |  |  | **Low** | **Low** | **Low** |
| **Adjustment for other prognostic factors** | | |  |  |  |  |  |  |  |
| a. Measurement of all other important PFs measured | | | |  |  |  | No | No | No |
| b. Provision of clear definitions of important PFs measured | | | | |  |  | Yes | Yes | Moderate |
| c. Adequately valid and reliable measurement of all important PFs | | | | |  |  | Yes | Yes | Yes |
| d. Use of same method and setting of PFs measurement in all study participants | | | | | |  | Yes | Yes | Yes |
| e. Appropriate imputation methods used for missing PFs | | | | |  |  | Moderate | No | Moderate |
| f. Important PFs were accounted for in the study design | | | |  |  |  | No | Yes | Yes |
| g. Important PFs were accounted for in the analysis | | | |  |  |  | No | Yes | Yes |
| **Risk of bias** |  |  |  |  |  |  | **High** | **Low** | **Low** |
| **Statistical analysis and reporting** | | |  |  |  |  |  |  |  |
| a. Sufficient presentation of data to assess the adequacy of the analytic strategy | | | | | |  | Yes | Yes | Yes |
| b. Strategy for model building is appropriate and based on a conceptual framework or model | | | | | | | Moderate | Yes | Yes |
| c. Statistical model is adequate for the study design | | | |  |  |  | Yes | Yes | Yes |
| d. No selective reporting of results | | |  |  |  |  | Yes | Moderate | Moderate |
| **Risk of bias** |  |  |  |  |  |  | **Moderate** | **Moderate** | **Moderate** |
| **Overall risk of bias** | |  |  |  |  |  | **HIGH** | **MODERATE** | **MODERATE** |

**Supplementary 5. QUIPS template cont.**

|  |  |  |  |  |  |  | **Serrano-Villar 2022** | **Martínez-Sanz 2023** |
| --- | --- | --- | --- | --- | --- | --- | --- | --- |
| **Study participation** | |  |  |  |  |  |  |  |
| a. Adequate participation in the study by eligible persons | | | | |  |  | Yes | Yes |
| b. Description of the target population or population of interest | | | | |  |  | Yes | Yes |
| c. Description of the baseline study sample | | | |  |  |  | Yes | Yes |
| d. Adequate description of the sampling frame and recruitment | | | | |  |  | Yes | Yes |
| e. Adequate description of the period and place of recruitment | | | | |  |  | Yes | Yes |
| f. Adequate description of inclusion and exclusion criteria | | | | |  |  | No | No |
| **Risk of bias** |  |  |  |  |  |  | **Low** | **Low** |
| **Study attrition** | |  |  |  |  |  |  |  |
| a. Adequate response rate for study participants | | | |  |  |  | Yes | Yes |
| b. Description of attempts to collect information on participants who dropped out | | | | | |  | Yes | No |
| c. Reasons for loss to follow‐up provided | | |  |  |  |  | No | No |
| d. Adequate description of participants lost to follow‐up | | | |  |  |  | Yes | No |
| e. No important differences between participants who completed the study and those who did not | | | | | | | Moderate | Moderate |
| **Risk of bias** |  |  |  |  |  |  | **Low** | **High** |
| **Prognostic factor measurement** | | |  |  |  |  |  |  |
| a. Clear definition or description provided | | |  |  |  |  | Yes | Yes |
| b. Adequately valid and reliable method of measurement | | | | |  |  | Yes | Yes |
| c. Continuous variables reported or appropriate cut points used | | | | |  |  | Yes | Yes |
| d. Same method and setting of measurement used in all study participants | | | | | |  | Yes | Yes |
| e. Appropriate methods of imputation were used for missing data | | | | |  |  | Yes | Yes |
| **Risk of bias** |  |  |  |  |  |  | **Low** | **Low** |
| **Outcome measurement** | |  |  |  |  |  |  |  |
| a. Clear definition of the outcome provided | | | |  |  |  | Yes | Yes |
| b. Adequately valid and reliable method of outcome measurement | | | | |  |  | Yes | Yes |
| c. Same method and setting of outcome measurement used in all study participants | | | | | |  | Yes | Yes |
| **Risk of bias** |  |  |  |  |  |  | **Low** | **Low** |
| **Adjustment for other prognostic factors** | | |  |  |  |  |  |  |
| a. Measurement of all other important PFs measured | | | |  |  |  | No | No |
| b. Provision of clear definitions of important PFs measured | | | | |  |  | Yes | Yes |
| c. Adequately valid and reliable measurement of all important PFs | | | | |  |  | Yes | Yes |
| d. Use of same method and setting of PFs measurement in all study participants | | | | | |  | Yes | Yes |
| e. Appropriate imputation methods used for missing PFs | | | | |  |  | Yes | Yes |
| f. Important PFs were accounted for in the study design | | | |  |  |  | Yes | Yes |
| g. Important PFs were accounted for in the analysis | | | |  |  |  | Yes | Yes |
| **Risk of bias** |  |  |  |  |  |  | **Low** | **Low** |
| **Statistical analysis and reporting** | | |  |  |  |  |  |  |
| a. Sufficient presentation of data to assess the adequacy of the analytic strategy | | | | | |  | Yes | Yes |
| b. Strategy for model building is appropriate and based on a conceptual framework or model | | | | | | | Yes | Yes |
| c. Statistical model is adequate for the study design | | | |  |  |  | Yes | Yes |
| d. No selective reporting of results | | |  |  |  |  | Moderate | Moderate |
| **Risk of bias** |  |  |  |  |  |  | **Moderate** | **Moderate** |
| **Overall risk of bias** | |  |  |  |  |  | **LOW** | **MODERATE** |

**Supplementary 6. Adapted Grading of Recommendations, Assessment, Development and Evaluation (GRADE) for systematic reviews with meta-analysis of prognostic studies**

**Supplementary 7. Primary studies reporting Non-AIDS events**

| **Non-AIDS event** | **Total of studies** |
| --- | --- |
| Non-AIDS cancer | 13 |
| Cardiovascular | 12 |
| Kidney | 10 |
| Hepatic | 8 |
| Cerebrovascular | 7 |
| Bacterial infections | 5 |
| Diabetes mellitus | 4 |
| Dyslipidemia | 2 |
| COPD | 2 |
| Osteoporosis | 2 |


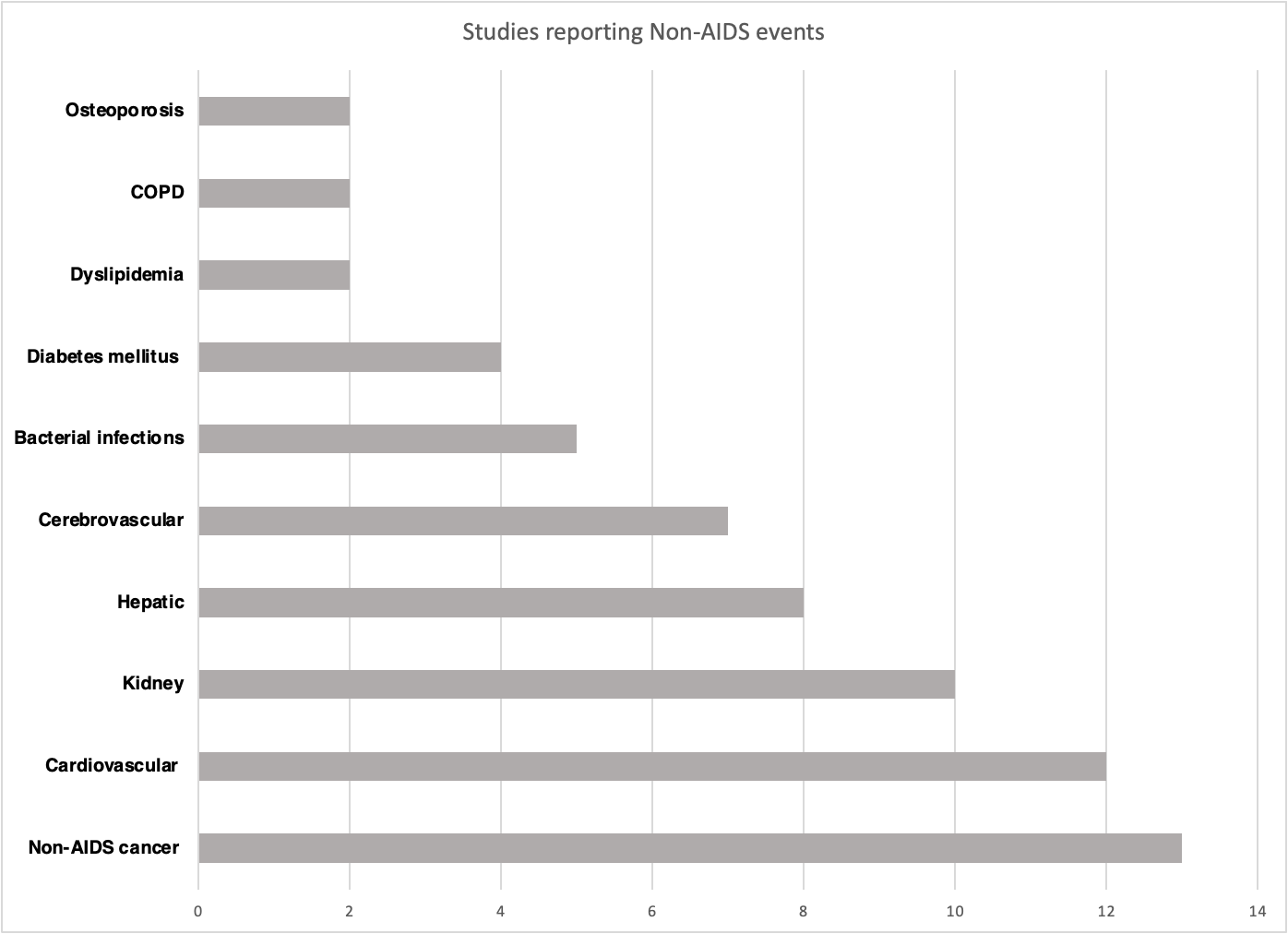

Supplement: Supplementary file 1 [file DataSheet_1.docx]
